# Supplementary material for: Regio- and chemoselective catalytic partial transfer hydrogenation of quinolines by dinuclear aluminum species
Source: Nat Commun. 2025 Sep 5;16:8230. doi: 10.1038/s41467-025-63460-9 (PMC12413467; doi:10.1038/s41467-025-63460-9)
Supplement: Supplementary file 1 — Supplementary Information [file 41467_2025_63460_MOESM1_ESM.pdf]

Supplementary Information

Regio- and chemoselective catalytic partial transfer hydrogenation of quinolines by dinuclear aluminum species

Contents

1. Supplementary Methods ... 3

1.1 General Methods and Instrumentation ... 3

1.2 Reaction Development ... 4

1.2.1 General procedure for catalytic 1,2-reduction of quinolines ... 4

1.2.2 Optimization studies ... 4

1.2.3 Reaction profile ... 6

1.3 Synthesis and Characterization ... 7

1.3.1 Synthesis of Al-2 ... 7

1.3.2 Synthesis of Al-3 ... 9

1.3.3 Synthesis of Al-4 ... 11

1.3.4 Characterization data for products 2a-2l ... 13

1.4 Mechanistic Studies ... 25

1.4.1 Competitive reaction between quinoline and H_3NBH_3 ... 25

1.4.2 Stoichiometric reactions of Al-2(or Al-3) with quinoline or H_3NBH_3 ... 26

1.4.3 Deuterium-labelling experiments ... 29

1.4.4 KIE experiments ... 31

1.4.5 Kinetic experiments ... 31

2 Single Crystal X-Ray Structure Determination ... 34

3 Computational Details ... 39

4 Supplementary References ... 44

1. Supplementary Methods

1.1 General Methods and Instrumentation

All experiments and manipulations were carried out under argon atmosphere using standard Schlenk or glovebox techniques. The glassware was heat-dried under vacuum prior to use. All glass junctions were coated with PTFE-based grease Merkel Triboflon III. For stirring, PTFE-coated magnetic stirrer bars were used or glass-coated ones if stated. Liquid phases were transferred using standard PE/PP syringes equipped with stainless steel cannula or directly canted from vessel to vessel if not stated otherwise. Solvents were dried by standard methods (withdrawal from MBraun Solvent Purification System and storage over molecular sieves, or distilled from sodium/ benzophenone or CaH_2 under argon atmosphere and degassed via freeze-pump-thaw cycling). All chemicals were purchased from commercial suppliers and used as received if not stated otherwise. Deuterated solvents were obtained from Deutero Deutschland GmbH and were dried over molecular sieves. All NMR samples were prepared under argon in J. Young PTFE tubes. NMR spectra were recorded on a Bruker AV400US, DRX400, AVHD300 and AV500cr at ambient temperature (300 K) if not stated otherwise. ^{1}H and ^{13}C NMR spectra were calibrated against the residual proton and natural abundance carbon resonances of the respective deuterated solvent as internal standard. Liquid Injection Field Desorption Ionization Mass Spectrometry (LIFDI-MS) was measured directly from an inert atmosphere glovebox with a Thermo Fisher Scientific Exactive Plus Orbitrap equipped with an ion source from Linden CMS. ATR-FTIR spectra were recorded on a Perkin Elmer FTIR spectrometer (diamond ATR, Spectrum Two; located inside an argon-filled glovebox) in a range of 400 – 4000 cm^{-1}.

1.2 Reaction Development

1.2.1 General procedure for catalytic 1,2-reduction of quinolines

In glovebox, aluminum species (5 mol%), ammonia borane (0.1−0.2 mmol), quinoline substrate (0.1 mmol) and C_6D_6 (0.5 mL) were added sequentially to a 4 mL vial. The reaction mixture was stirred at the indicated temperature (25−40 °C) and was monitored by ^1H NMR. After completion, the resulting solution was concentrated in vacuum and the residue was purified by silica gel column chromatography (EtOAc/hexane) to give the hydrogenated product.

1.2.2 Optimization studies

Supplementary Table 1. Optimization of reaction conditions for the transfer-hydrogenation of **1a**.

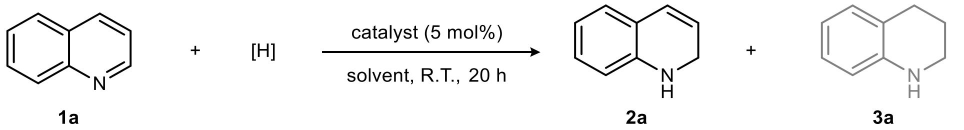

entry	catalyst	[H]	solvent	yield of 2a[a]	2a : 3a[b]
1	none	H_3NBH_3 (2 eq.)	C_6D_6	< 5%	-
2	**Al-1**	Me_2HNBH_3 (2 eq.)	C_6D_6	< 5%	-
3	**Al-1**	H_3NBH_3 (2 eq.)	C_6D_6	86%	6 : 1
4	**Al-1**	H_3NBH_3 (2 eq.)	Tol-d_8	62%	8 : 1
5	**Al-1**	H_3NBH_3 (2 eq.)	THF-d_8	75%	3 : 1
6	**Al-1**	H_3NBH_3 (1 eq.)	C_6D_6	94%	16 : 1
7	$AlCl_3$	H_3NBH_3 (1 eq.)	C_6D_6	< 5%	-
8	Al^tBu_3	H_3NBH_3 (1 eq.)	C_6D_6	9%	>20 : 1
9	$Al(Si^tBu_2Me)_3$	H_3NBH_3 (1 eq.)	C_6D_6	15%	>20 : 1
10	NHC•AlH_3	H_3NBH_3 (1 eq.)	C_6D_6	66%	8 : 1
11	NMe_3•AlH_3	H_3NBH_3 (1 eq.)	C_6D_6	9%	>20 : 1

[a]The yield was determined by crude ^1H NMR analysis of the reaction mixture with 1,3,5-trimethoxybenzene as internal standard. [b]The ratio was determined by crude ^1H NMR analysis of the reaction mixture.

Supplementary Table 2. TON and TOF examination of **Al-1**.

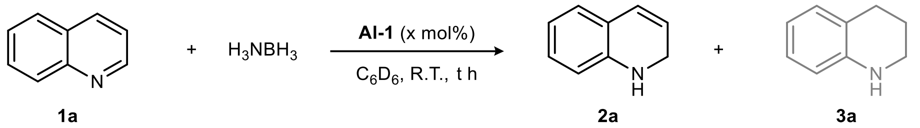

entry	x mol%	t (h)	yield of 2a[a]	2a : 3a[b]	TON	TOF (h^{-1})
1	5 mol%	20 h	94%	16 : 1	19	1 h^{-1}
2	2 mol%	20 h	94%	>20 : 1	47	2.4 h^{-1}
3	1 mol%	20 h	89%	20 : 1	89	4.5 h^{-1}
4	0.5 mol%	48 h	80%	16 : 1	160	3.3 h^{-1}

[a]The yield was determined by crude ^1H NMR analysis of the reaction mixture with 1,3,5-trimethoxybenzene as internal standard. [b]The ratio was determined by crude ^1H NMR analysis of the reaction mixture.

Supplementary Table 3. TON and TOF examination of **Al-2** and **Al-3**.

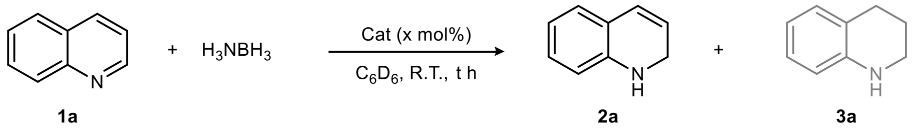

entry	Cat	x mol%	t (h)	yield of 2a[a]	2a : 3a[b]	TON	TOF (h^{-1})
1	**Al-2**	5 mol%	20 h	94%	18 : 1	19	1 h^{-1}
2	**Al-2**	2 mol%	20 h	61%	14 : 1	31	1.6 h^{-1}
3	**Al-3**	5 mol%	20 h	93%	18 : 1	19	1 h^{-1}
4	**Al-3**	1 mol%	20 h	88%	>20 : 1	88	4.4 h^{-1}
5	**Al-3**	0.5 mol%	48 h	89%	18 : 1	178	3.7 h^{-1}

[a]The yield was determined by crude ^1H NMR analysis of the reaction mixture with 1,3,5-trimethoxybenzene as internal standard. [b]The ratio was determined by crude ^1H NMR analysis of the reaction mixture.

1.2.3 Reaction profile

The hydrogenation process was monitored by ^{1}H NMR under standard conditions and a kinetic profile was shown as follows:

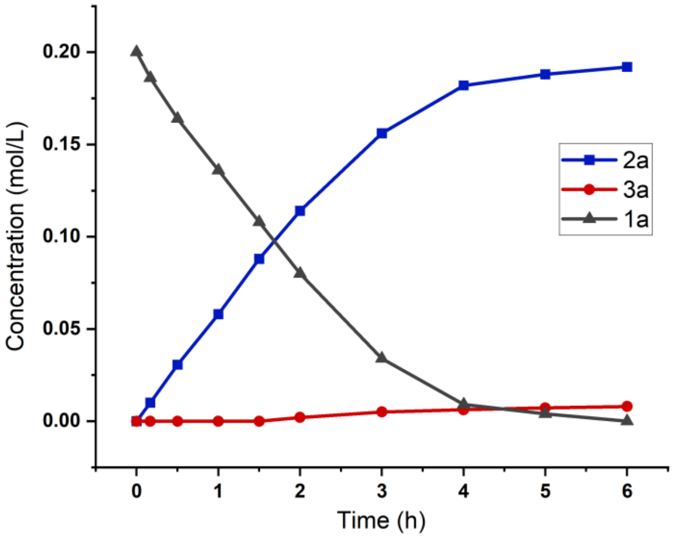

Supplementary Figure 1. Kinetic profile for the catalytic 1,2-reduction of **1a**.

1.3 Synthesis and Characterization

1.3.1 Synthesis of Al-2

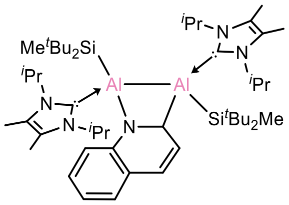

A mixture of dialumene **Al-1** (30 mg, 0.041 mmol) and quinoline (5.3 mg, 0.041 mmol) was stirred in benzene (1 mL) at room temperature for 2 hours. The color of the solution changed from dark purple to yellow. All volatiles were dried under reduced pressure, and the residue was washed with cold pentane (2 × 0.1 mL) to give **Al-2** as a yellow powder (19.7 mg, 56% yield).

^{1}H NMR (400 MHz, C_6D_6): δ [ppm] 6.99 (td, *J* = 7.6, 1.7 Hz, 1H, C*H*-quinoline), 6.67 – 6.57 (m, 2H, C*H*-quinoline), 6.43 (td, *J* = 7.2, 1.1 Hz, 1H, C*H*-quinoline), 6.29 – 6.21 (m, 1H, C*H*(CH_3)$_2$), 6.19 (dd, *J* = 9.5, 2.6 Hz, 1H, C*H*-quinoline), 5.96 – 5.82 (m, 2H, C*H*(CH_3)$_2$ overlapping with C*H*-quinoline), 4.98 – 4.87 (m, 1H, C*H*(CH_3)$_2$), 4.76 – 4.72 (m, 1H, C*H*-Al), 4.72 – 4.64 (m, 1H, C*H*(CH_3)$_2$), 1.86 (d, *J* = 6.8 Hz, 3H, CH(C*H*$_3$)$_2$), 1.73 (d, *J* = 6.9 Hz, 3H, CH(C*H*$_3$)$_2$), 1.70 (s, 3H, *CH$_3$* NHC), 1.68 – 1.57 (m, 15H, *CH$_3$* NHC overlapping with CH(C*H*$_3$)$_2$), 1.39 – 1.33 (m, 9H, *CH$_3$* NHC overlapping with CH(C*H*$_3$)$_2$), 1.32 (s, 9H, C(C*H*$_3$)$_3$), 1.30 (d, *J* = 5.4 Hz, 3H, CH(C*H*$_3$)$_2$), 1.12 (s, 9H, C(C*H*$_3$)$_3$), 1.08 (s, 9H, C(C*H*$_3$)$_3$), 1.02 (s, 9H, C(C*H*$_3$)$_3$), 0.32 (s, 3H, SiC*H$_3$*), 0.26 (s, 3H, SiC*H$_3$*).

^{13}C{^{1}H} NMR (101 MHz, C_6D_6): δ [ppm] 153.54 (*C*=*C* quinoline), 136.44 (*C*=*C* quinoline), 126.89, 125.99, 125.97, 125.90, 125.58, 125.16, 124.38 (peaks between 124.3 and 126.9 are assigned as *C*=*C* quinoline and *C*=*C* NHC), 119.39 (*C*=*C* quinoline), 115.81 (*C*=*C* quinoline), 113.55 (*C*=*C* quinoline), 53.82 (*C*H(CH_3)$_2$), 51.53 (*C*H(CH_3)$_2$), 51.20 (*C*H(CH_3)$_2$), 50.76 (*C*H(CH_3)$_2$), 31.67 (C(*C*H$_3$)$_3$), 31.07 (C(*C*H$_3$)$_3$), 30.59 (C(*C*H$_3$)$_3$), 30.34 (C(*C*H$_3$)$_3$), 22.83, 22.58, 22.51, 22.35, 22.23, 22.07, 21.92, 21.83, 21.60, 21.38, 21.07 (peaks between 21.0 and 23.5 are assigned as Si*C*(CH_3)$_3$ and CH(*C*H$_3$)$_2$), 9.91 (*C*H$_3$ NHC), 9.82 (*C*H$_3$ NHC), 9.73 (*C*H$_3$ NHC), 9.72 (*C*H$_3$ NHC), -2.88 (Si*C*H$_3$), -4.01 (Si*C*H$_3$).

LIFDI-MS: $C_{49}H_{89}Al_2N_5Si_2$, Calcd: 857.6287; Found: 857.6248.

No signal was found for aluminum-bonded silicon and carbene carbon atom in the ^{29}Si{^{1}H}, ^{13}C{^{1}H} NMR spectrum respectively, due to the quadrupolar momentum of the ^{27}Al nucleus.

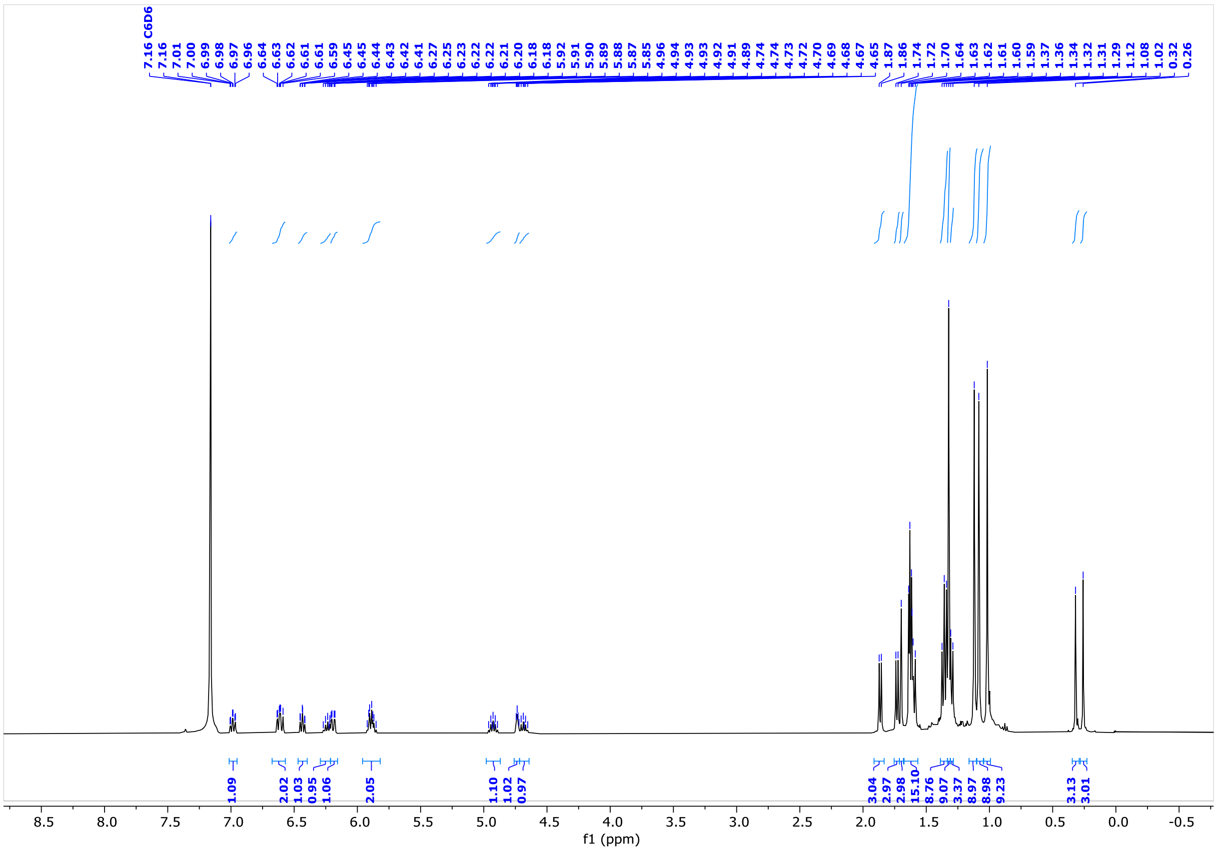

Supplementary Figure 2. ^{1}H NMR spectrum of **Al-2** in C_6D_6 at 300K.

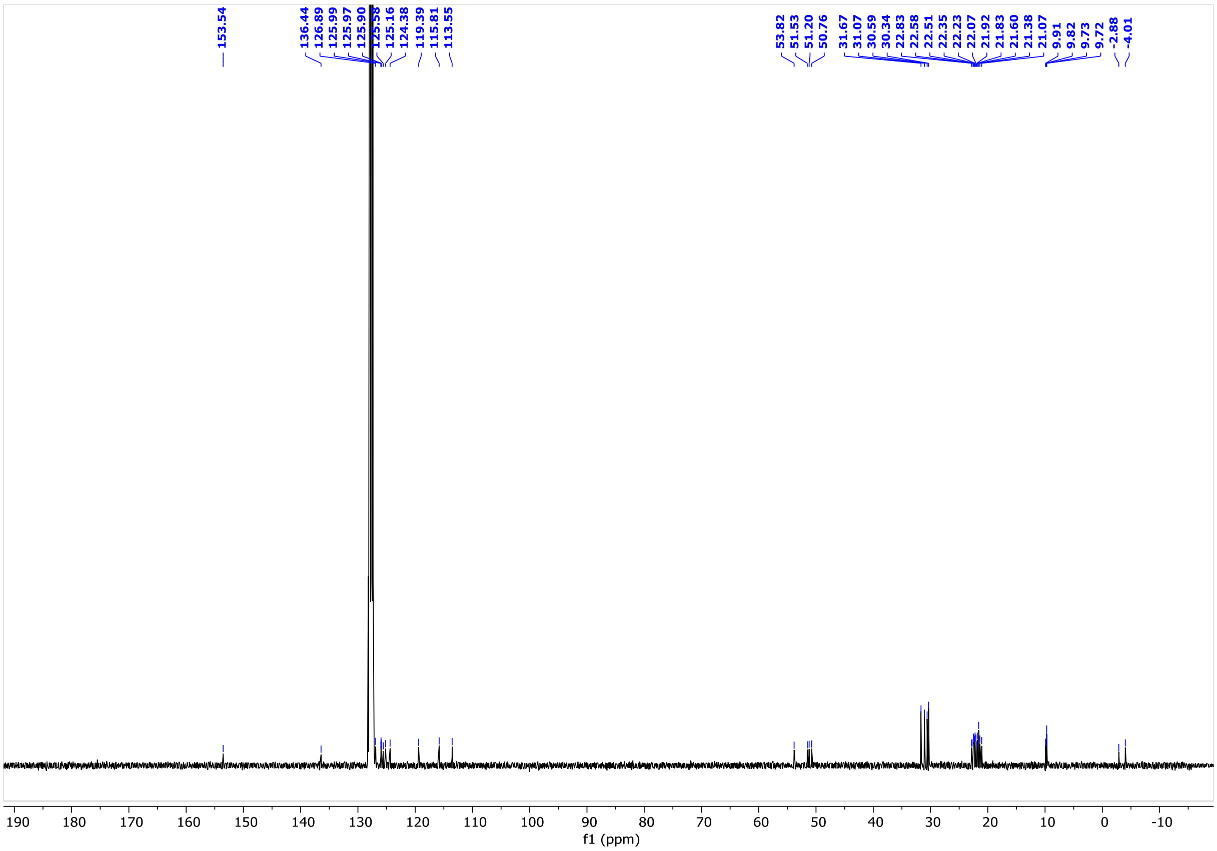

Supplementary Figure 3. $^{13}C\{^{1}H\}$ NMR spectrum of **Al-2** in C_6D_6 at 300K.

1.3.2 Synthesis of Al-3

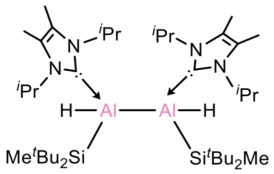

A mixture of dialumene **Al-1** (30 mg, 0.041 mmol) and H_3NBH_3 (1.3 mg, 0.041 mmol) was stirred in benzene (1 mL) at room temperature for 2 hours. The color of the solution changed from dark purple to light yellow. All volatiles were dried under reduced pressure, and the residue was washed with cold pentane (2 × 0.2 mL) to give **Al-3** as a colorless powder (12 mg, 41% yield).

^{1}H NMR (400 MHz, C_6D_6): δ [ppm] 6.76 – 4.98 (m, 4H, C*H*(CH_3)$_2$), 4.43 (br, 2H, Al-*H*), 1.60 (s, 12H, *CH$_3$* NHC), 1.58 (s, 18H, C(C*H*$_3$)$_3$), 1.40 (s, 18H, C(C*H*$_3$)$_3$), 1.35 – 1.28 (m, 12H, CH(C*H*$_3$)$_2$), 1.27 – 1.03 (m, 12H, CH(C*H*$_3$)$_2$), 0.35 (s, 6H, SiC*H$_3$*).

^{13}C{^{1}H} NMR (101 MHz, C_6D_6): δ [ppm] 179.09 (carbene atom NHC), 125.41(*C*=*C* NHC), 51.96 (*C*H(CH_3)$_2$), 31.66 (C(*C*H$_3$)$_3$), 31.16 (C(*C*H$_3$)$_3$), 22.15 (br, CH(*C*H$_3$)$_2$), 21.84 (Si*C*(CH_3)$_3$), 21.75 (Si*C*(CH_3)$_3$), 9.96 (*C*H$_3$ NHC), -3.74 (Si*C*H$_3$).

LIFDI-MS: $C_{40}H_{84}Al_2N_4Si_2$, Calcd: 730.5865; Found: 729.5778 ([**Al-3**]–H).

No signal was found for aluminum-bonded silicon atom in the ^{29}Si{^{1}H} NMR spectrum, due to the quadrupolar momentum of the ^{27}Al nucleus.

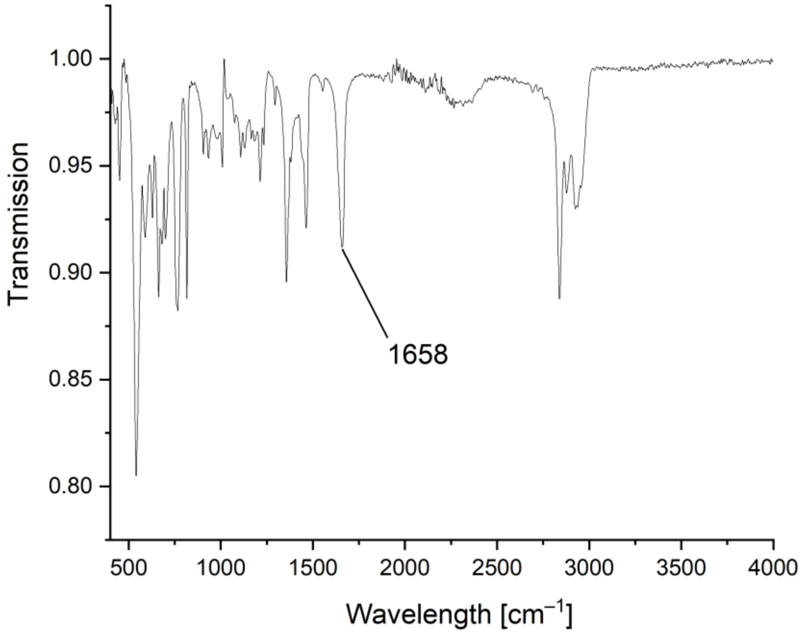

Supplementary Figure 4. IR data of complex **Al-3**.

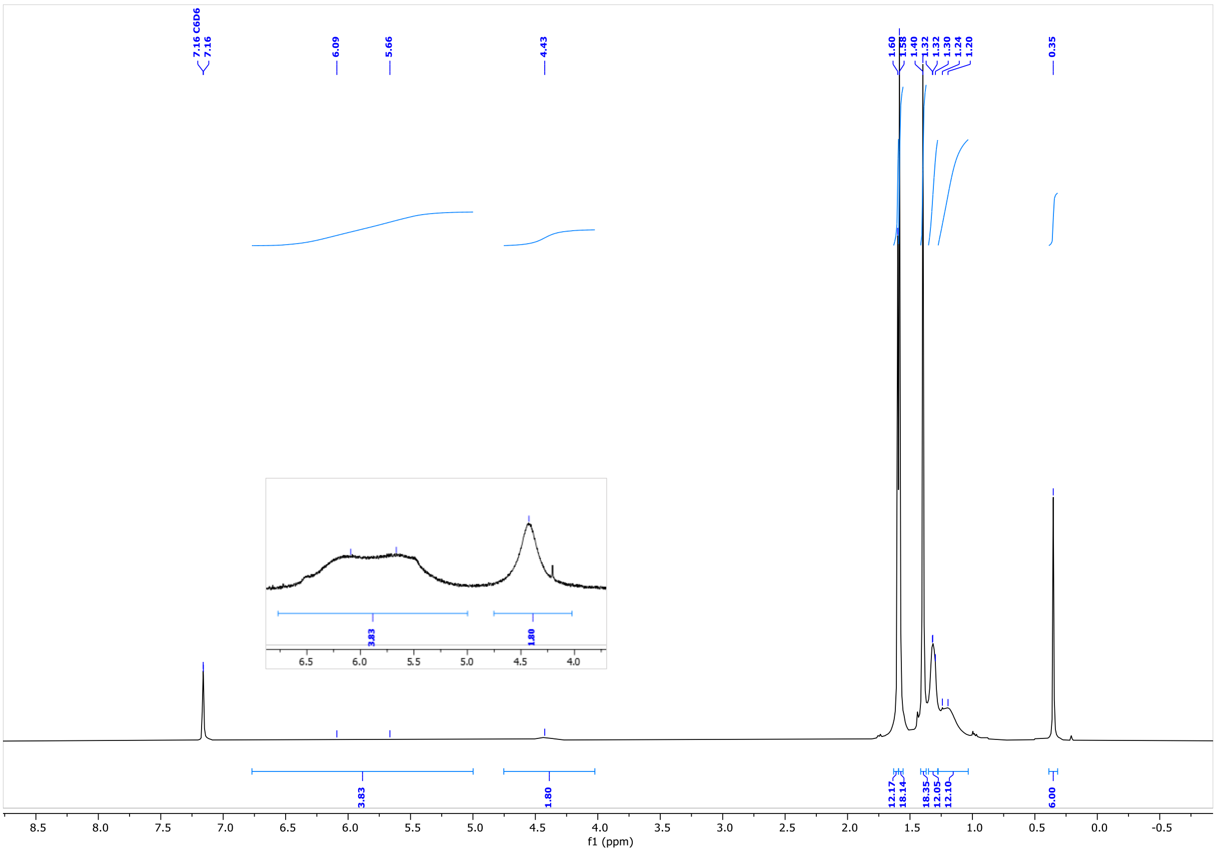

Supplementary Figure 5. ^{1}H NMR spectrum of **Al-3** in C_6D_6 at 300K.

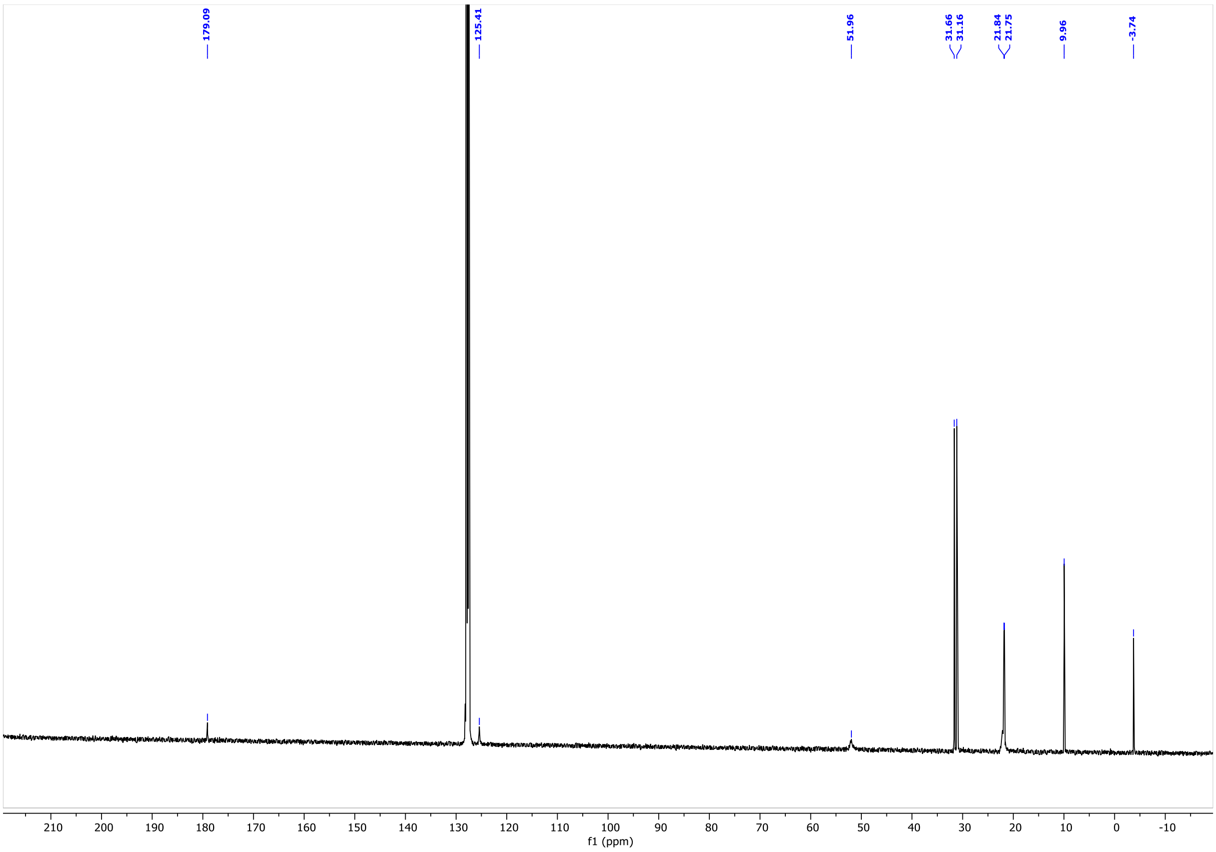

Supplementary Figure 6. $^{13}C\{^{1}H\}$ NMR spectrum of **Al-3** in C_6D_6 at 300K.

1.3.3 Synthesis of Al-4

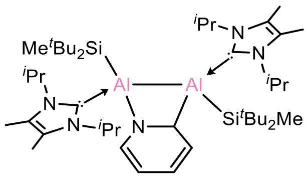

A mixture of dialumene **Al-1** (30 mg, 0.041 mmol) and pyridine (3.3 mg, 0.041 mmol) was stirred in benzene (0.5 mL) at room temperature for 2 hours. The color of the solution changed from dark purple to orange-red. All volatiles were dried under reduced pressure, and the residue was recrystallized in toluene at -30 °C to give **Al-4** as an orange powder (15 mg, 45% yield).

^{1}H NMR (400 MHz, C_6D_6): δ [ppm] 6.77 (d, *J* = 6.7 Hz, 1H, C*H*-pyridine), 6.40 (hept, *J* = 6.8 Hz, 1H, C*H*(CH_3)$_2$), 6.23 (hept, *J* = 6.8 Hz, 1H, C*H*(CH_3)$_2$), 5.78 (dt, *J* = 8.9, 4.2 Hz, 1H, C*H*-pyridine), 5.35 (d, *J* = 9.5 Hz, 1H, C*H*-pyridine), 4.98 (hept, *J* = 7.0 Hz, 1H, C*H*(CH_3)$_2$), 4.75 – 4.69 (m, 1H, C*H*-Al), 4.69 – 4.57 (m, 2H, C*H*(CH_3)$_2$ overlapping with C*H*-pyridine), 2.15 – 2.08 (m, 6H, CH(C*H*$_3$)$_2$), 1.79 (s, 3H, *CH$_3$* NHC), 1.72 (s, 3H, *CH$_3$* NHC), 1.66 (s, 3H, *CH$_3$* NHC), 1.62 (s, 3H, *CH$_3$* NHC), 1.58 – 1.53 (m, 6H, CH(C*H*$_3$)$_2$), 1.41 (d, *J* = 7.1 Hz, 3H, CH(C*H*$_3$)$_2$), 1.36 (d, *J* = 7.0 Hz, 6H, CH(C*H*$_3$)$_2$), 1.31 (d, *J* = 7.1 Hz, 3H, CH(C*H*$_3$)$_2$), 1.18 (s, 9H, C(C*H*$_3$)$_3$), 1.15 (s, 18H, C(C*H*$_3$)$_3$), 1.05 (s, 9H, C(C*H*$_3$)$_3$), 0.25 (s, 3H, SiC*H*$_3$), 0.22 (s, 3H, SiC*H*$_3$).

^{13}C{^{1}H} NMR (101 MHz, C_6D_6): δ [ppm] 176.44 (*carbene* NHC), 175.19 (*carbene* NHC), 147.27 (*C*=*C* pyridine), 126.21, 125.97, 125.92, 125.48, 121.82, 120.06 (peaks between 126.3 and 120.0 are assigned as *C*=*C* pyridine and *C*=*C* NHC), 92.32 (*C*=*C* pyridine), 54.80 (*C*H(CH_3)$_2$), 51.82 (*C*H(CH_3)$_2$), 51.44 (*C*H(CH_3)$_2$), 51.35 (*C*H(CH_3)$_2$), 31.47 (C(*C*H$_3$)$_3$), 31.28 (C(*C*H$_3$)$_3$), 31.09 (C(*C*H$_3$)$_3$), 30.70 (C(*C*H$_3$)$_3$), 24.01, 23.09, 22.86, 22.70, 22.65, 22.44, 22.41, 21.98, 21.82, 21.69, 21.13, 21.12 (peaks between 24.1 and 21.0 are assigned as Si*C*(CH_3)$_3$ and CH(*C*H$_3$)$_2$), 10.31 (*C*H$_3$ NHC), 10.26 (*C*H$_3$ NHC), 10.20 (*C*H$_3$ NHC), 10.15 (*C*H$_3$ NHC), -3.96 (Si*C*H$_3$), -4.31 (Si*C*H$_3$).

LIFDI-MS: $C_{45}H_{87}Al_2N_5Si_2$, Calcd: 807.6131; Found: 728.5737 ([**Al-4**]–pyridine).

No signal was found for aluminum-bonded silicon and carbene carbon atom in the ^{29}Si{^{1}H}, ^{13}C{^{1}H} NMR spectrum respectively, due to the quadrupolar momentum of the ^{27}Al nucleus.

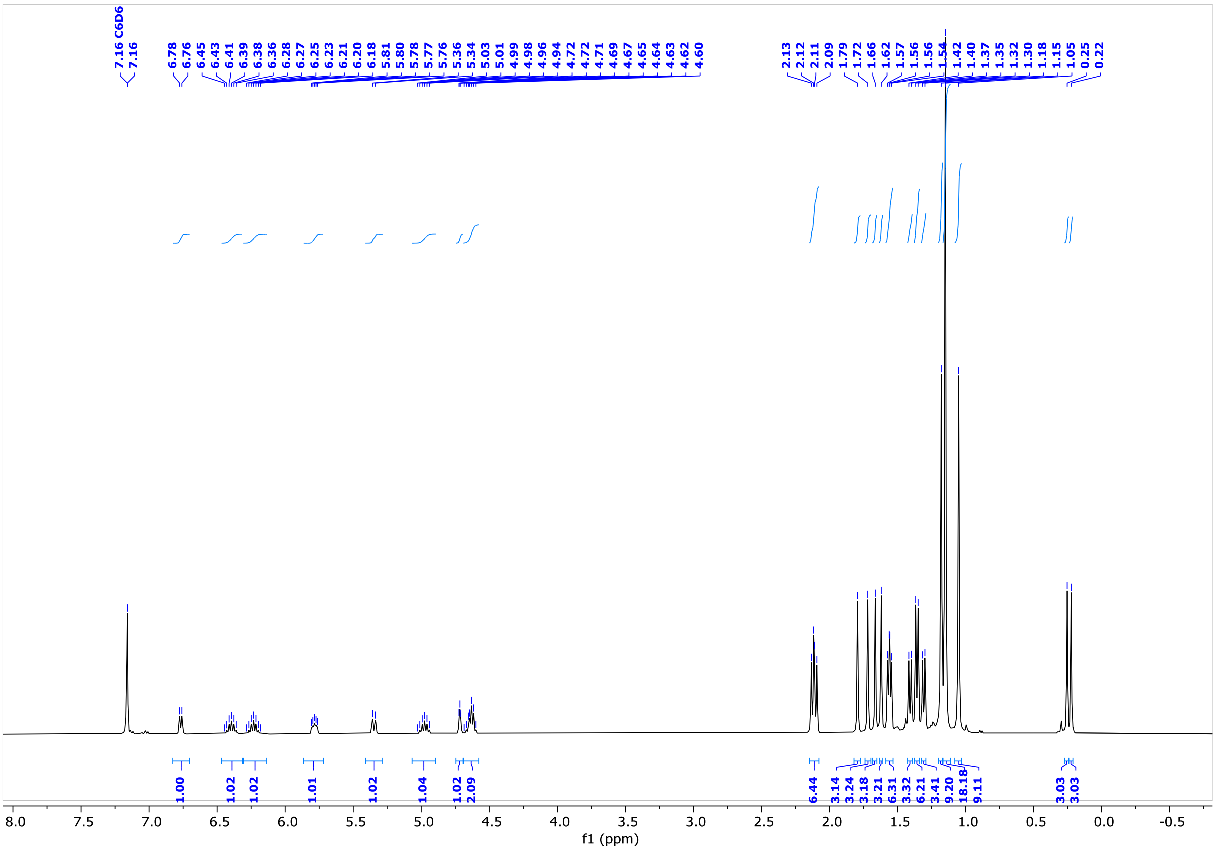

Supplementary Figure 7. ^{1}H NMR spectrum of **Al-4** in C_6D_6 at 300K.

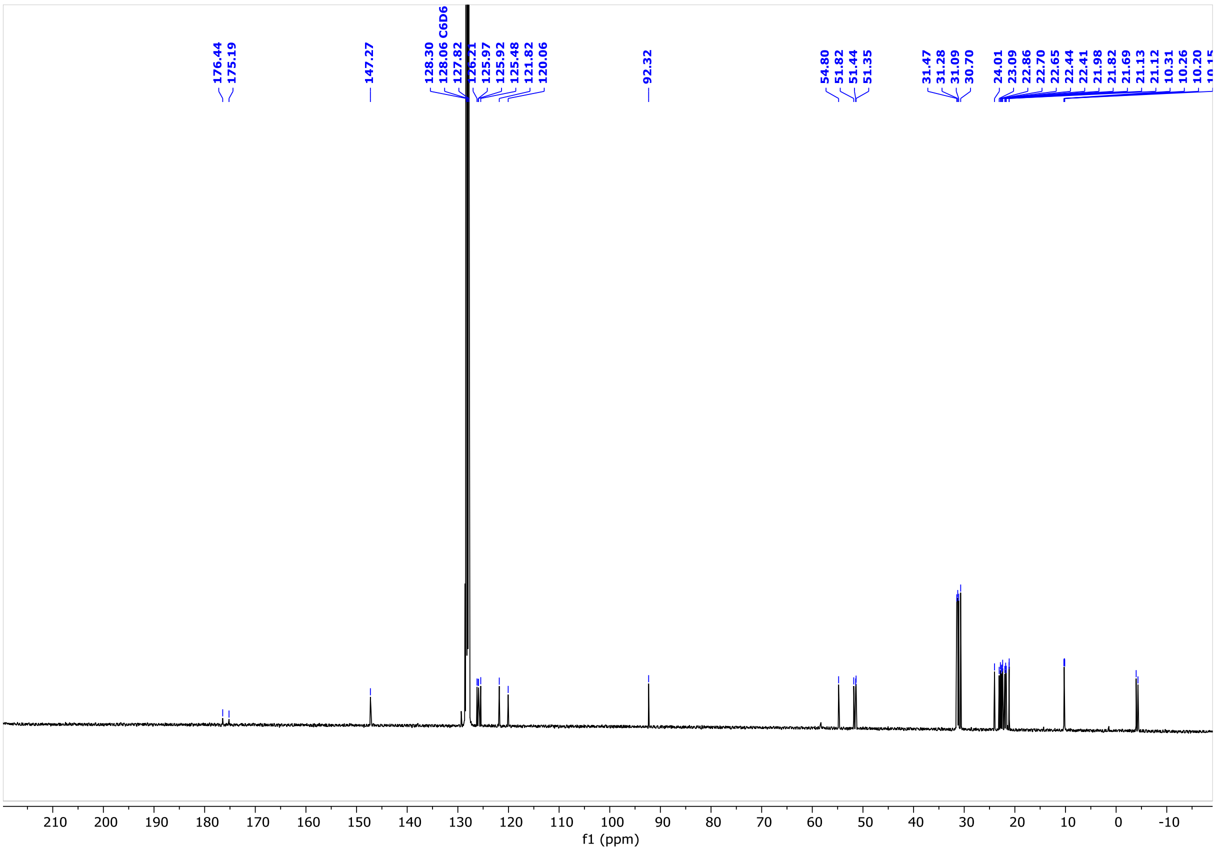

Supplementary Figure 8. $^{13}C\{^{1}H\}$ NMR spectrum of **Al-4** in C_6D_6 at 300K.

1.3.4 Characterization data for products 2a-2l

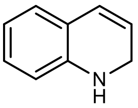

1,2-dihydroquinoline (2a): ^{1}H NMR (400 MHz, C_6D_6) δ 6.91 (td, *J* = 7.7, 1.6 Hz, 1H), 6.79 – 6.67 (m, 1H), 6.58 (td, *J* = 7.4, 1.1 Hz, 1H), 6.13 (d, *J* = 9.9 Hz, 1H), 6.04 (d, *J* = 7.9 Hz, 1H), 5.35 – 5.17 (m, 1H), 3.64 (dt, *J* = 3.8, 1.8 Hz, 2H), 2.59 (s, 1H). ^{13}C NMR (101 MHz, C_6D_6) δ 145.51, 128.95, 127.23, 126.83, 121.69, 121.17, 117.83, 112.71, 43.37.

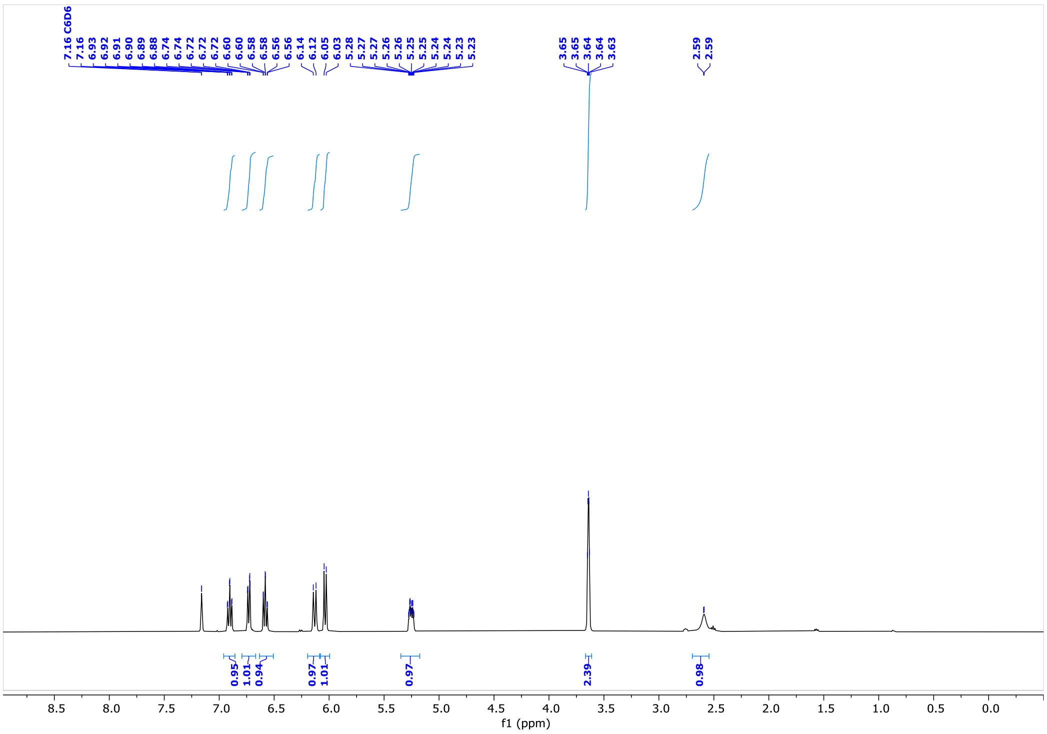

Supplementary Figure 9. ^{1}H NMR spectrum of **2a** in C_6D_6 at 300K.

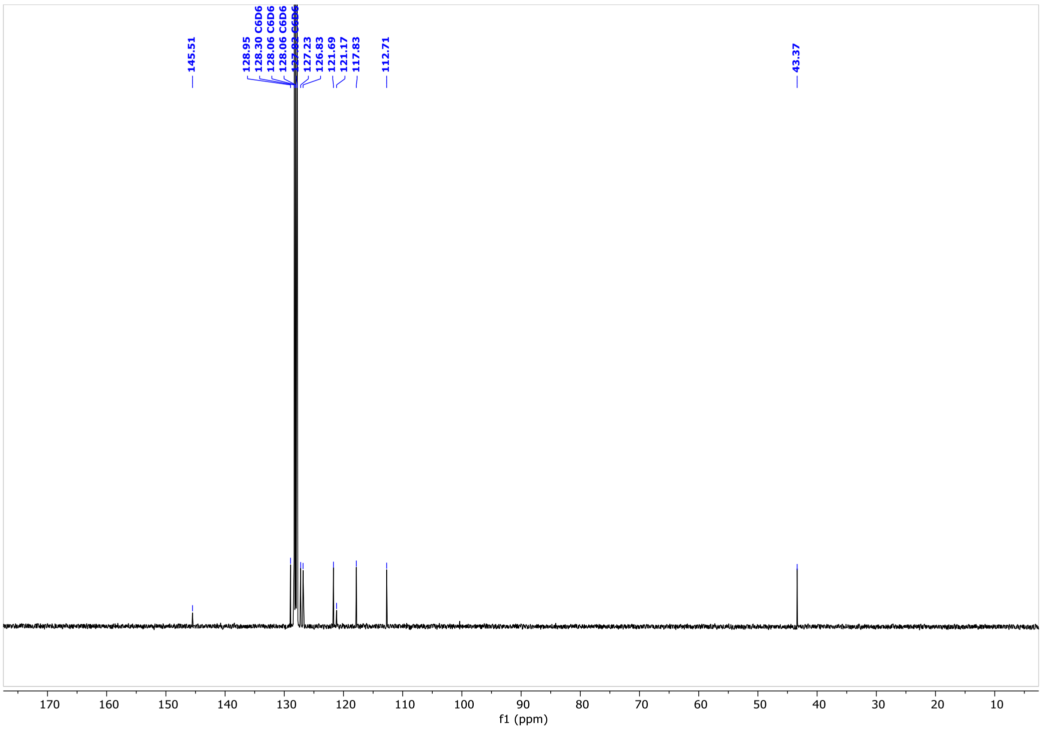

Supplementary Figure 10. ^{13}C{^{1}H} NMR spectrum of **2a** in C_6D_6 at 300K.

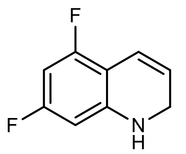

5,7-difluoro-1,2-dihydroquinoline (2b): ^{1}H NMR (400 MHz, C_6D_6) δ 6.41 (dt, *J* = 10.0, 2.1 Hz, 1H), 5.98 (td, *J* = 9.7, 2.4 Hz, 1H), 5.44 (dt, *J* = 10.5, 1.8 Hz, 1H), 5.10 – 4.96 (m, 1H), 3.40 (dt, *J* = 3.8, 1.8 Hz, 2H), 2.52 – 2.39 (m, 1H). ^{13}C NMR (101 MHz, C_6D_6) δ 163.31 (dd, *J* = 245.4, 16.2 Hz), 161.40 (dd, *J* = 246.4, 16.2 Hz), 146.94 (dd, *J* = 13.1, 10.1 Hz), 119.96 (t, *J* = 2.0 Hz), 117.70 (dd, *J* = 5.1, 2.0 Hz), 104.34 (dd, *J* = 18.2, 3.0 Hz), 95.08 (dd, *J* = 25.3, 3.0 Hz), 91.76 (t, *J* = 26.3 Hz), 42.44. ^{19}F NMR (377 MHz, C_6D_6) δ -111.24 (td, *J* = 10.0, 7.3 Hz), -121.58 (t, *J* = 8.7 Hz).

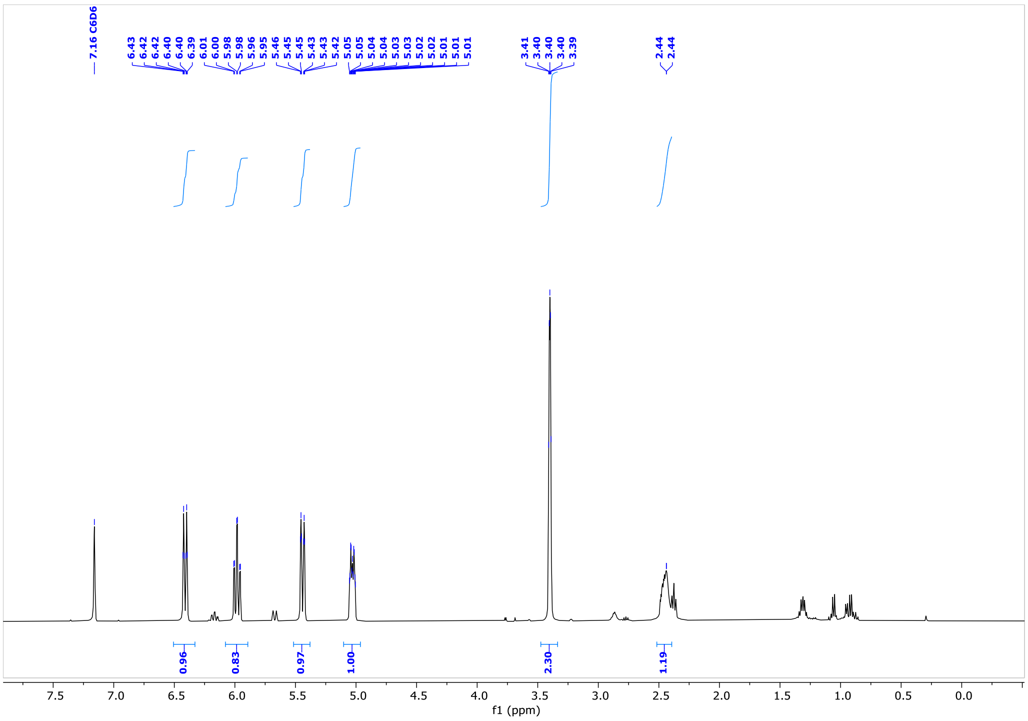

Supplementary Figure 11. ^{1}H NMR spectrum of **2b** in C_6D_6 at 300K.

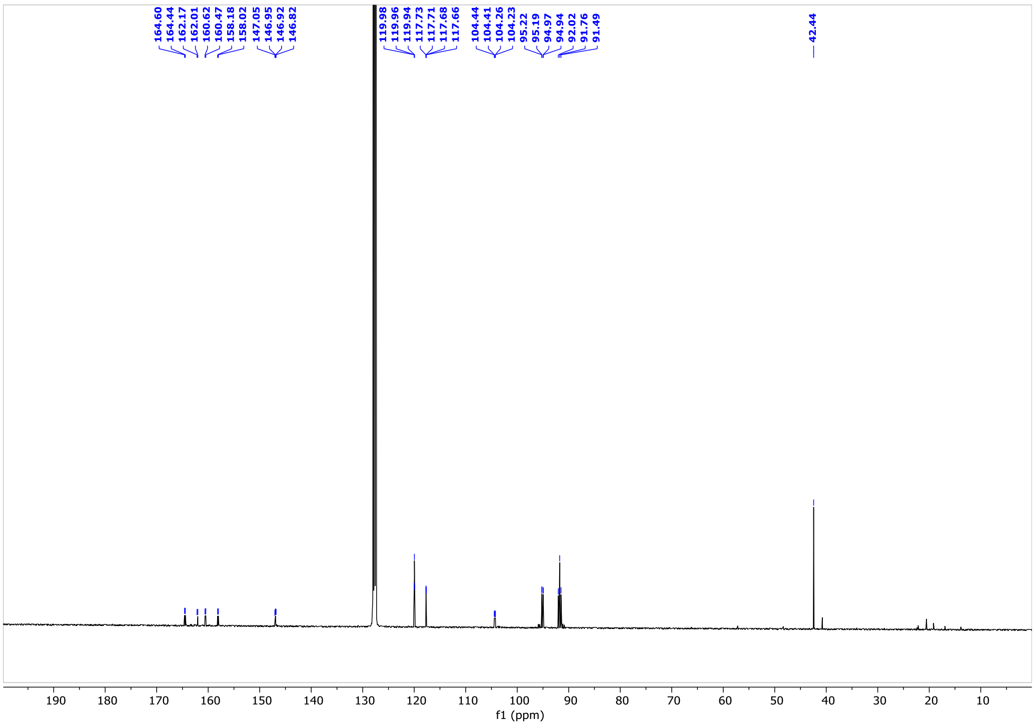

Supplementary Figure 12. ^{13}C{^{1}H} NMR spectrum of **2b** in C_6D_6 at 300K.

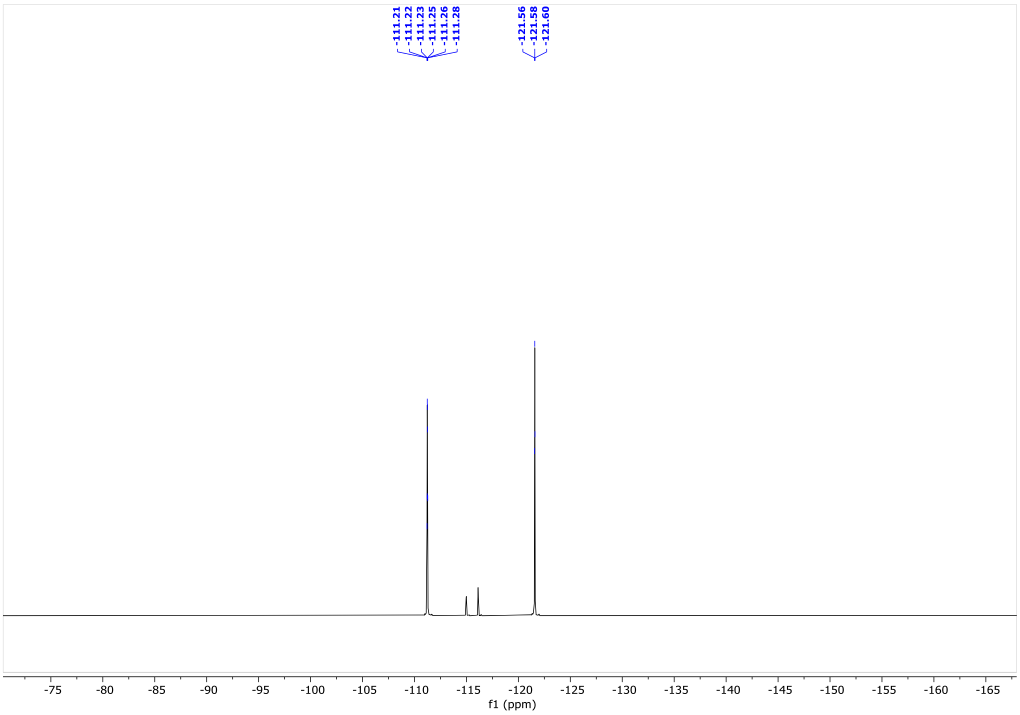

Supplementary Figure 13. $^{19}F\{^{1}H\}$ NMR spectrum of **2b** in C_6D_6 at 300K.

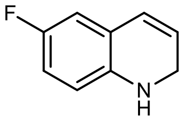

6-fluoro-1,2-dihydroquinoline (2c): ^{1}H NMR (400 MHz, C_6D_6) δ 6.59 (td, J = 8.7, 2.8 Hz, 1H), 6.45 (dd, J = 8.9, 2.9 Hz, 1H), 5.89 (dd, J = 9.8, 2.5 Hz, 1H), 5.76 (dd, J = 8.6, 4.5 Hz, 1H), 5.24 (dt, J = 9.3, 4.1 Hz, 1H), 3.51 (dt, J = 3.8, 1.8 Hz, 2H), 2.51 – 2.34 (m, 1H). ^{13}C NMR (101 MHz, C_6D_6) δ 156.44 (d, J = 234.3 Hz), 141.51, 126.00 (d, J = 2.0 Hz), 123.47, 122.22 (d, J = 8.1 Hz), 114.75 (d, J = 22.2 Hz), 113.49 (d, J = 23.2 Hz), 113.01 (d, J = 7.1 Hz), 43.29. ^{19}F NMR (377 MHz, C_6D_6) δ -128.12 (td, J = 8.7, 4.5 Hz).

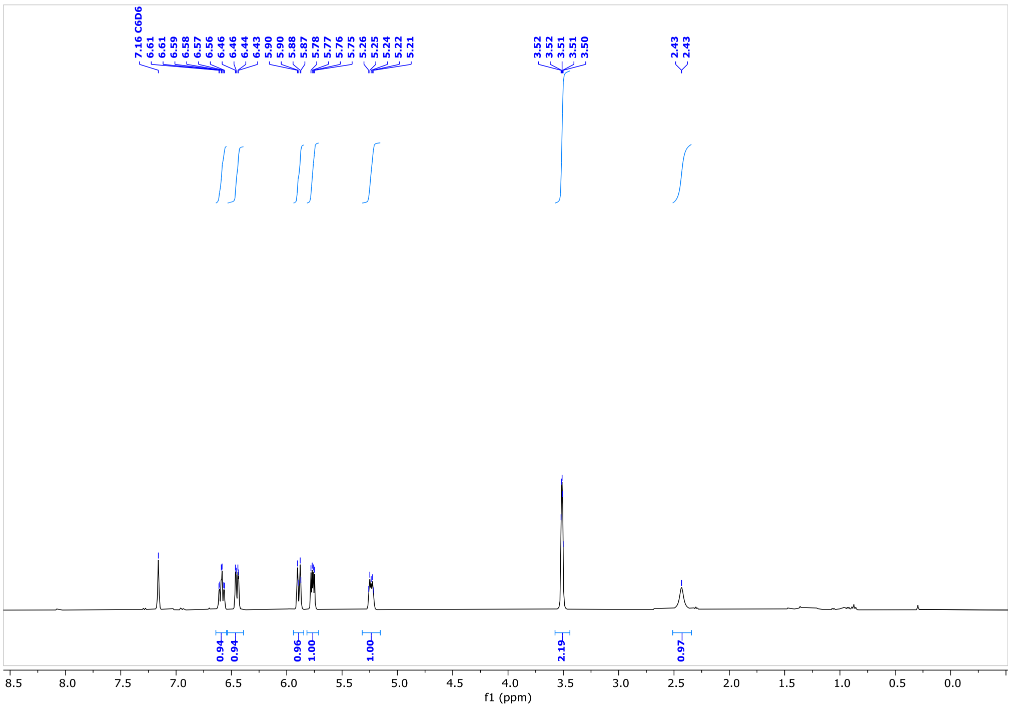

Supplementary Figure 14. ^{1}H NMR spectrum of **2c** in C_6D_6 at 300K.

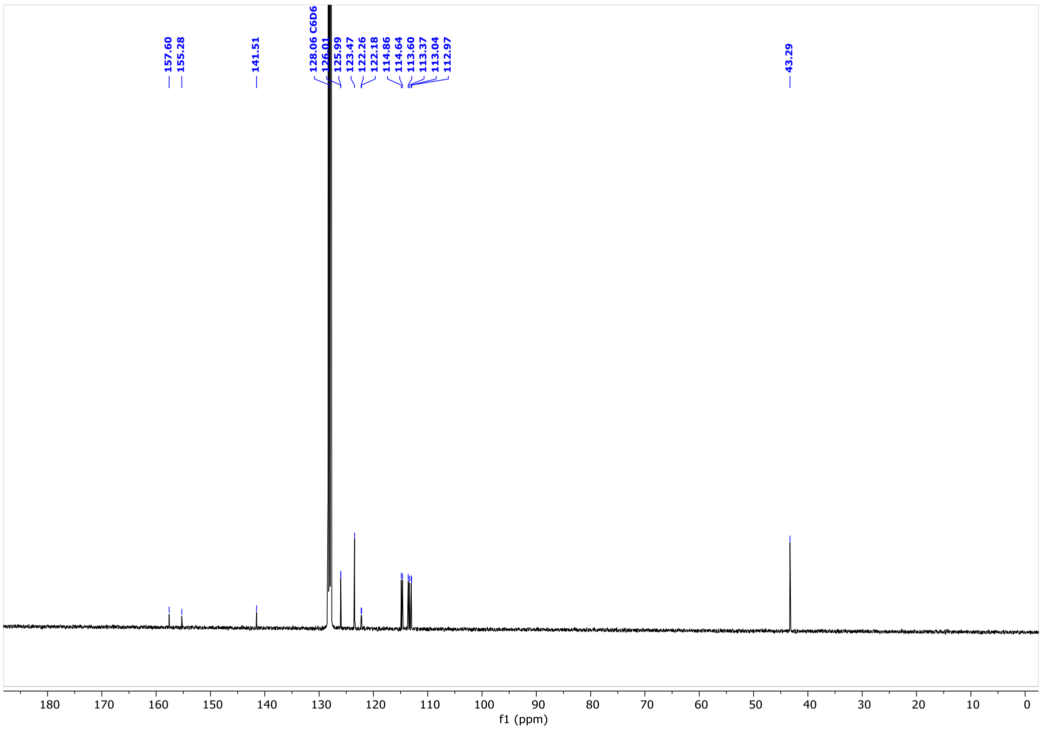

Supplementary Figure 15. $^{13}C\{^{1}H\}$ NMR spectrum of **2c** in C_6D_6 at 300K.

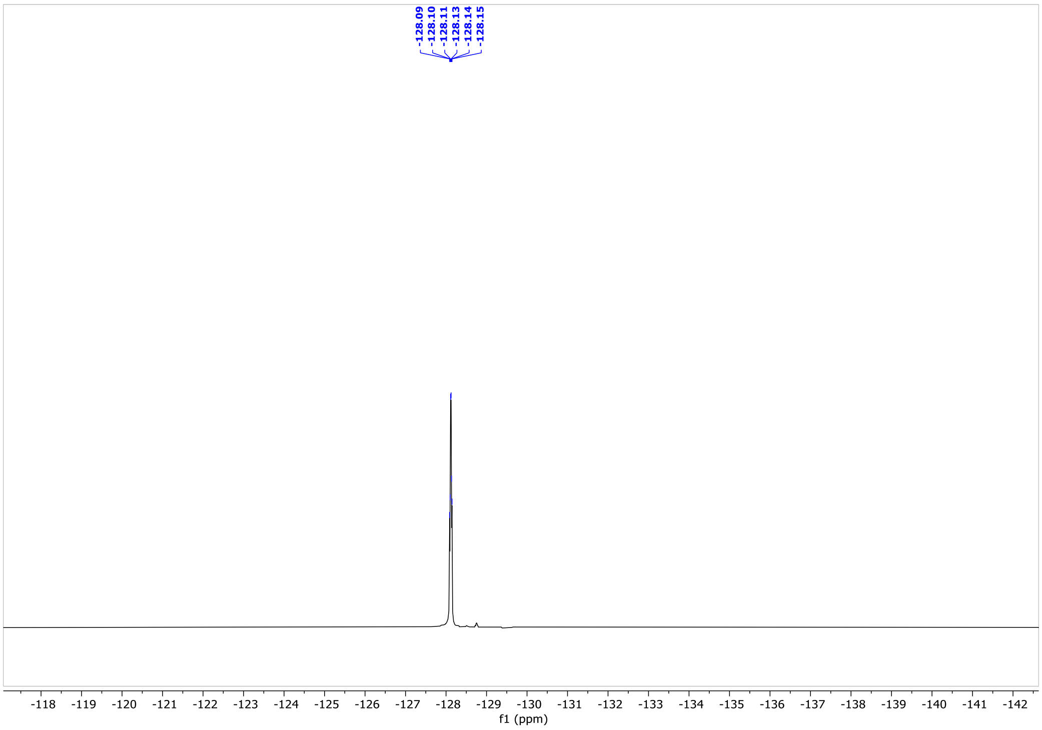

Supplementary Figure 16. $^{19}F\{^{1}H\}$ NMR spectrum of **2c** in C_6D_6 at 300K.

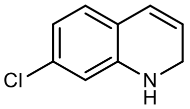

7-chloro-1,2-dihydroquinoline (2d): ^{1}H NMR (400 MHz, C_6D_6) δ 6.52 (dd, *J* = 7.9, 2.0 Hz, 1H), 6.37 (d, *J* = 7.9 Hz, 1H), 6.01 (d, *J* = 2.1 Hz, 1H), 5.93 (dt, *J* = 9.9, 2.1 Hz, 1H), 5.19 – 5.04 (m, 1H), 3.50 (dt, *J* = 3.7, 1.8 Hz, 2H), 2.41 (s, 1H). ^{13}C NMR (101 MHz, C_6D_6) δ 146.34, 134.15, 125.74, 121.64, 119.27, 117.38, 112.33, 43.12.

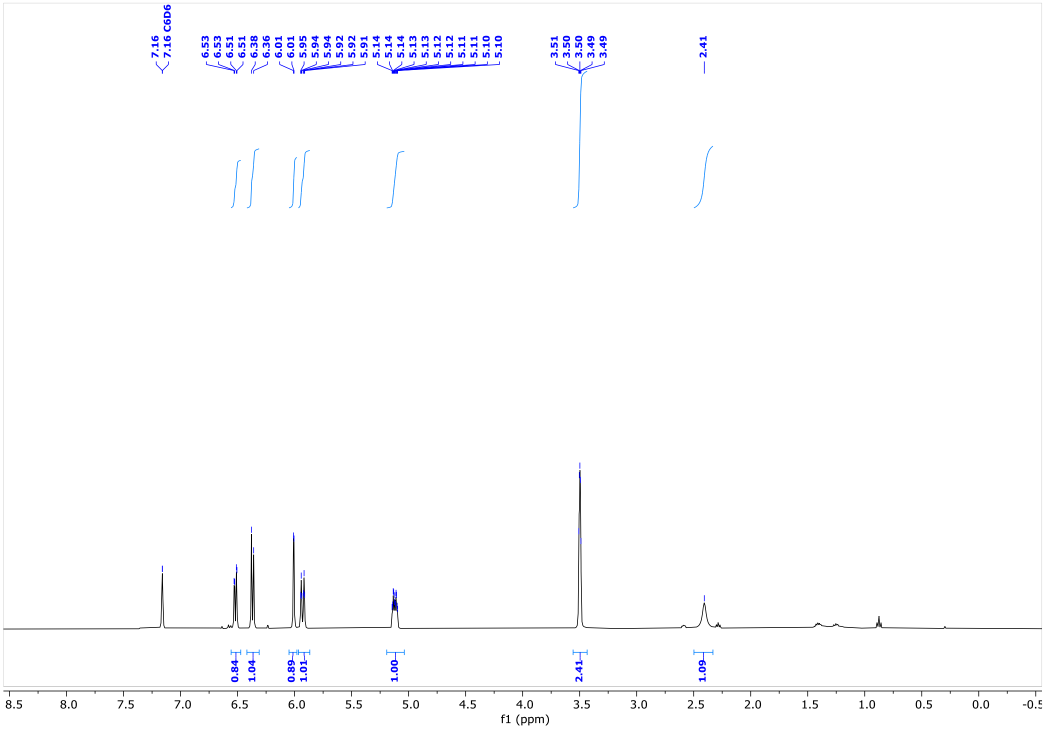

Supplementary Figure 17. ^{1}H NMR spectrum of **2d** in C_6D_6 at 300K.

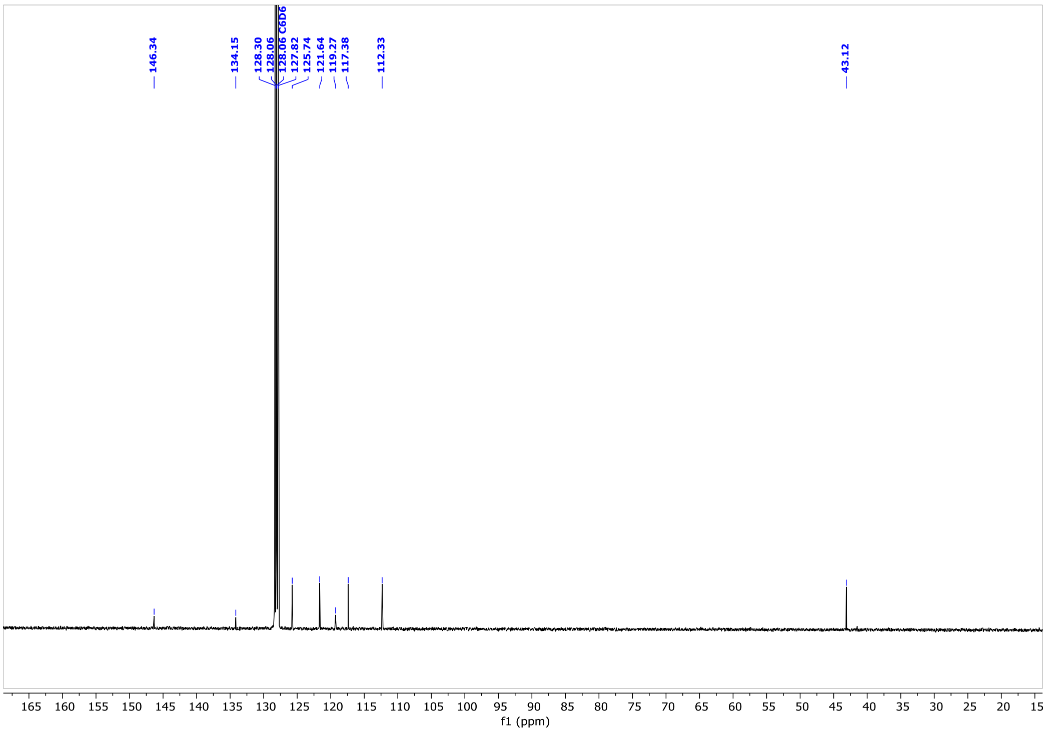

Supplementary Figure 18. ^{13}C{^{1}H} NMR spectrum of **2d** in C_6D_6 at 300K.

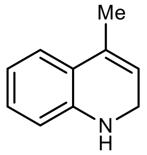

4-methyl-1,2-dihydroquinoline (2e): ^{1}H NMR (400 MHz, C_6D_6) δ 7.07 – 6.86 (m, 2H), 6.66 (td, J = 7.5, 1.2 Hz, 1H), 6.10 (dd, J = 8.2, 1.2 Hz, 1H), 5.15 (dt, J = 4.0, 2.0 Hz, 1H), 3.64 (dq, J = 3.6, 1.8 Hz, 2H), 2.69 (s, 1H), 1.77 (q, J = 1.7 Hz, 3H). ^{13}C NMR (101 MHz, C_6D_6) δ 145.74, 131.43, 128.79, 124.13, 122.70, 118.90, 117.68, 112.77, 43.27, 18.78.

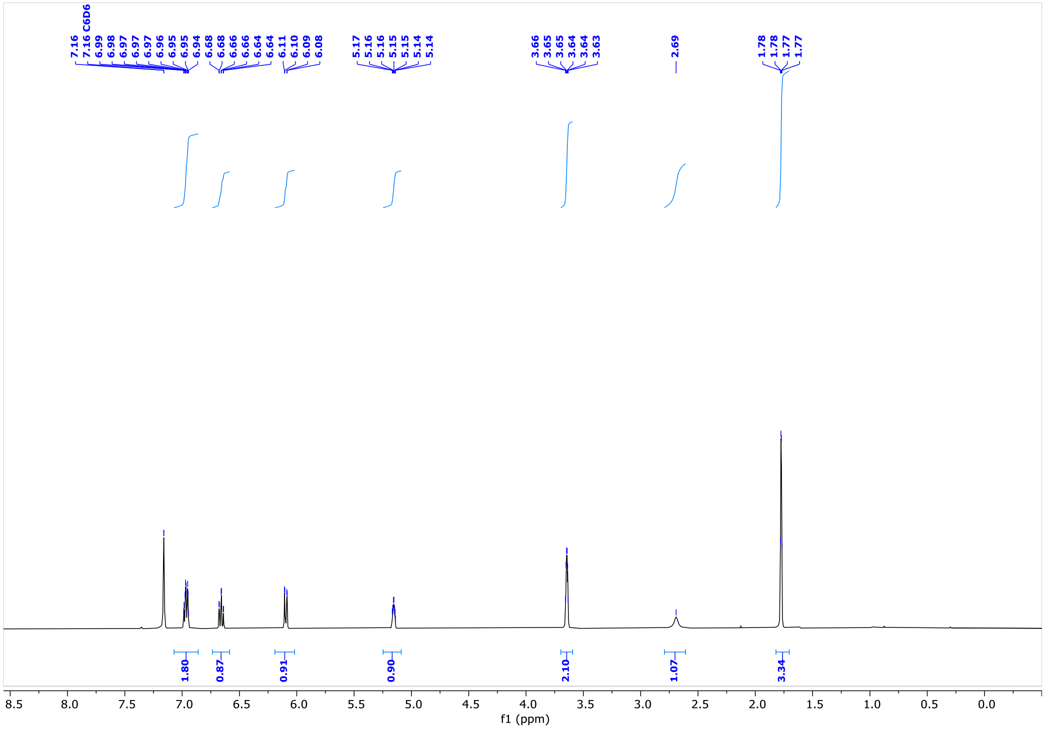

Supplementary Figure 19. ^{1}H NMR spectrum of **2e** in C_6D_6 at 300K.

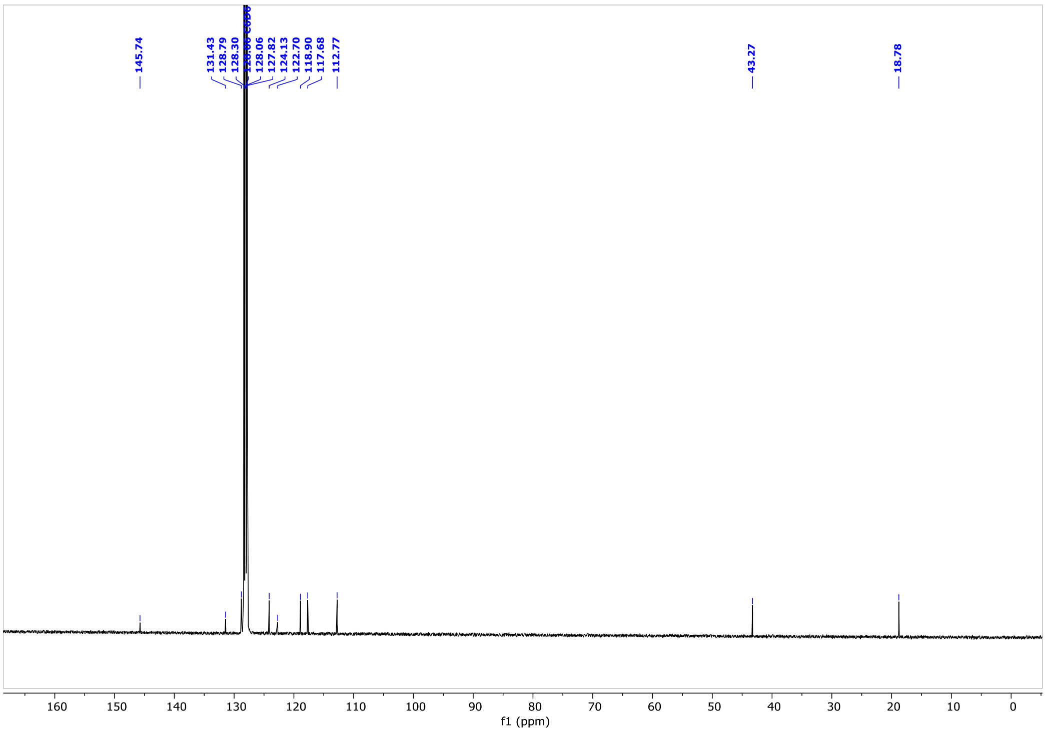

Supplementary Figure 20. ^{13}C{^{1}H} NMR spectrum of **2e** in C_6D_6 at 300K.

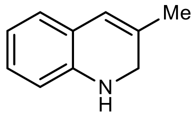

3-methyl-1,2-dihydroquinoline (2f): ^{1}H NMR (400 MHz, C_6D_6) δ 6.94 (td, *J* = 7.6, 1.6 Hz, 1H), 6.79 (dd, *J* = 7.3, 1.5 Hz, 1H), 6.65 (td, *J* = 7.4, 1.2 Hz, 1H), 6.12 (d, *J* = 7.8 Hz, 1H), 5.92 (q, *J* = 1.8 Hz, 1H), 3.57 (q, *J* = 1.5 Hz, 2H), 2.74 (s, 1H), 1.43 – 1.38 (m, 3H). ^{13}C NMR (101 MHz, C_6D_6) δ 143.79, 130.27, 126.39, 122.20, 121.79, 117.75, 112.21, 47.78, 20.80.

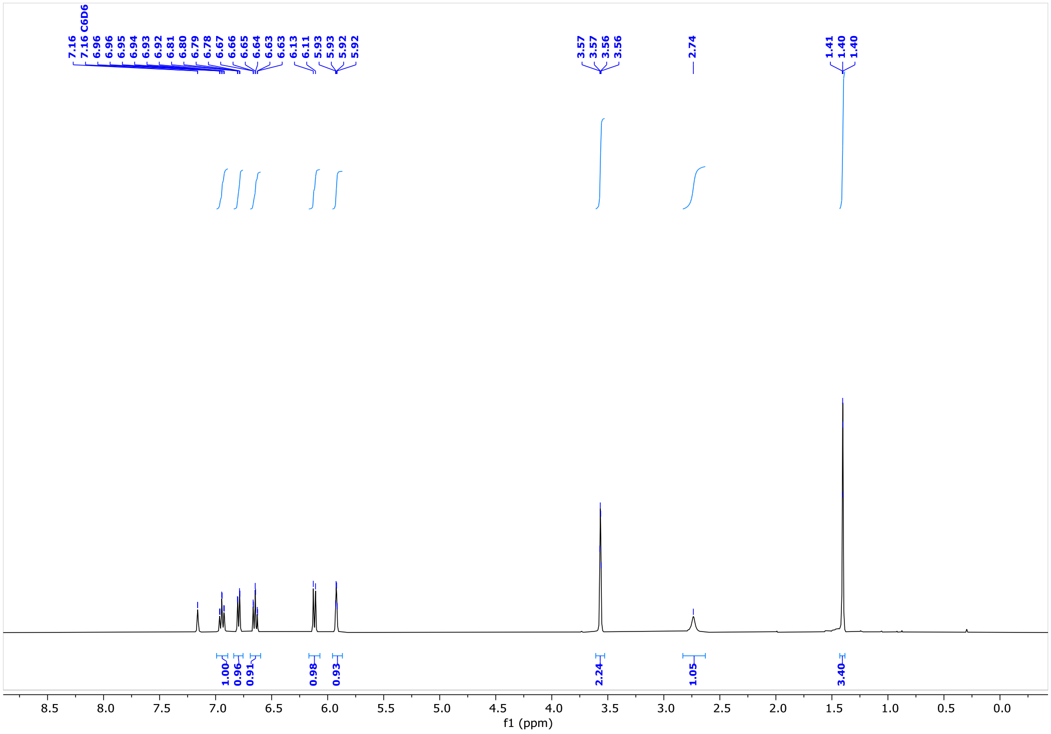

Supplementary Figure 21. ^{1}H NMR spectrum of **2f** in C_6D_6 at 300K.

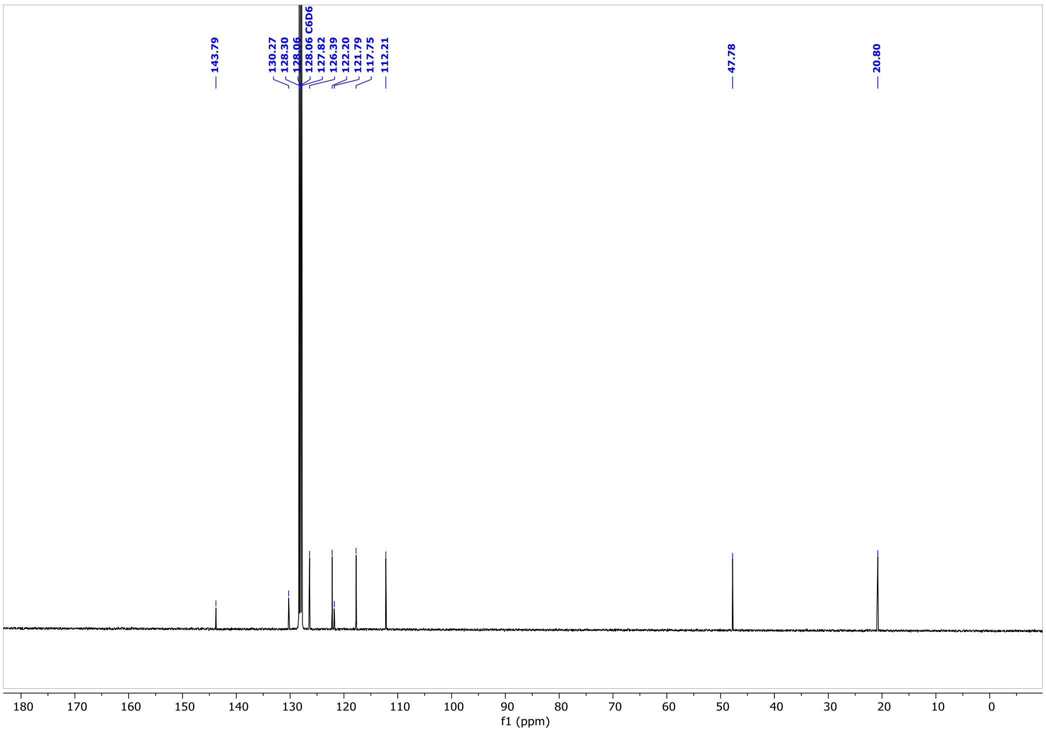

Supplementary Figure 22. $^{13}C\{^{1}H\}$ NMR spectrum of **2f** in C_6D_6 at 300K.

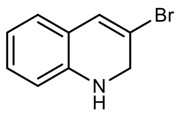

3-bromo-1,2-dihydroquinoline (2g): ^{1}H NMR (400 MHz, C_6D_6) δ 6.87 (dt, *J* = 7.9, 4.4 Hz, 1H), 6.52 (d, *J* = 4.3 Hz, 2H), 6.41 (d, *J* = 1.7 Hz, 1H), 5.95 (d, *J* = 7.9 Hz, 1H), 3.85 (t, *J* = 1.7 Hz, 2H), 2.42 (s, 1H). ^{13}C NMR (101 MHz, C_6D_6) δ 142.92, 129.23, 128.71, 126.86, 120.42, 118.10, 115.39, 112.74, 50.84.

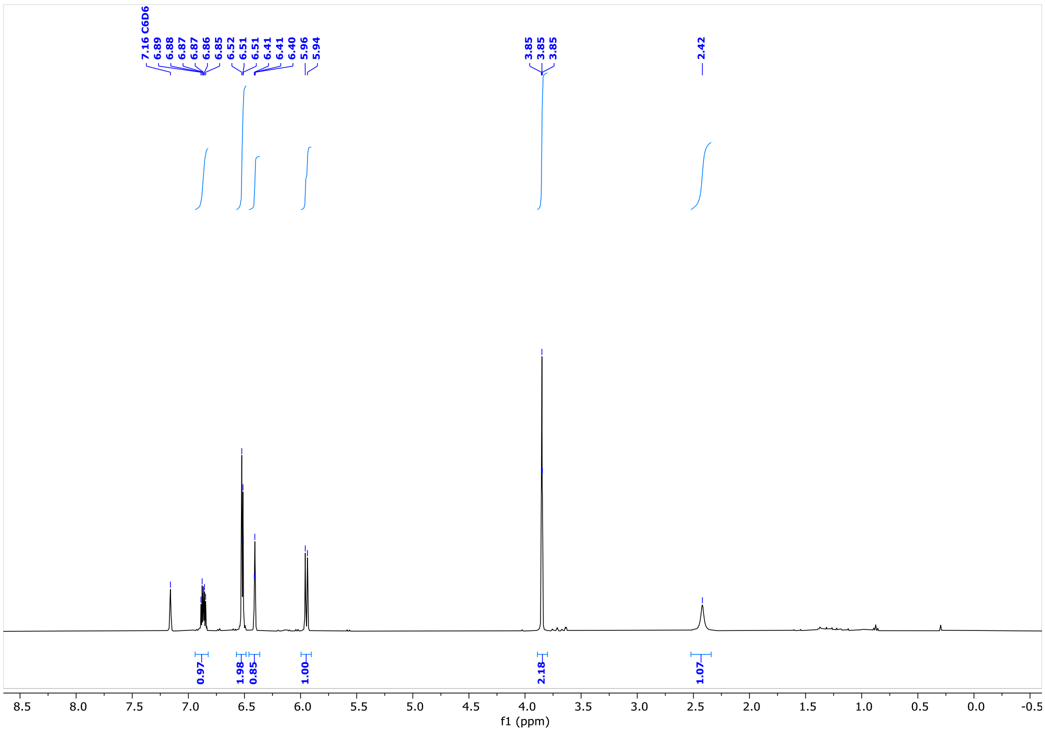

Supplementary Figure 23. ^{1}H NMR spectrum of **2g** in C_6D_6 at 300K.

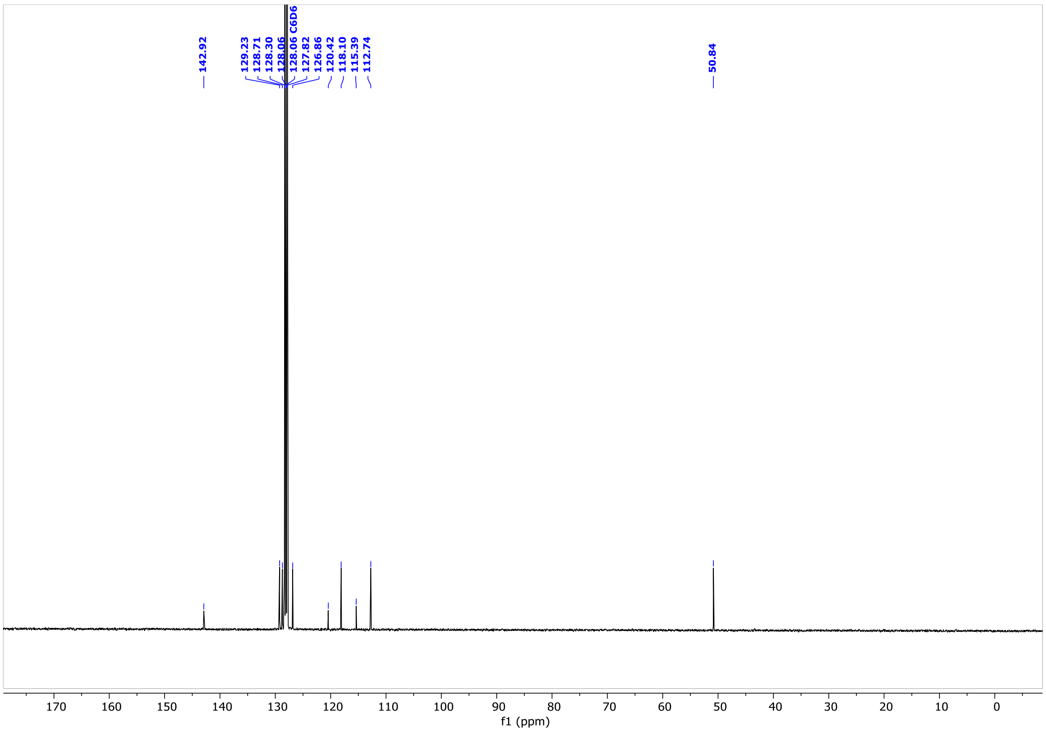

Supplementary Figure 24. $^{13}C\{^{1}H\}$ NMR spectrum of **2g** in C_6D_6 at 300K.

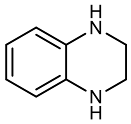

1,2,3,4-tetrahydroquinoxaline (2i): ^{1}H NMR (400 MHz, C_6D_6) δ 6.71 (dd, J = 5.8, 3.4 Hz, 2H), 6.32 (dd, J = 5.7, 3.4 Hz, 2H), 2.82 (s, 4H), 2.79 – 2.58 (m, 2H). ^{13}C NMR (101 MHz, C_6D_6) δ 134.17, 118.81, 114.82, 41.44.

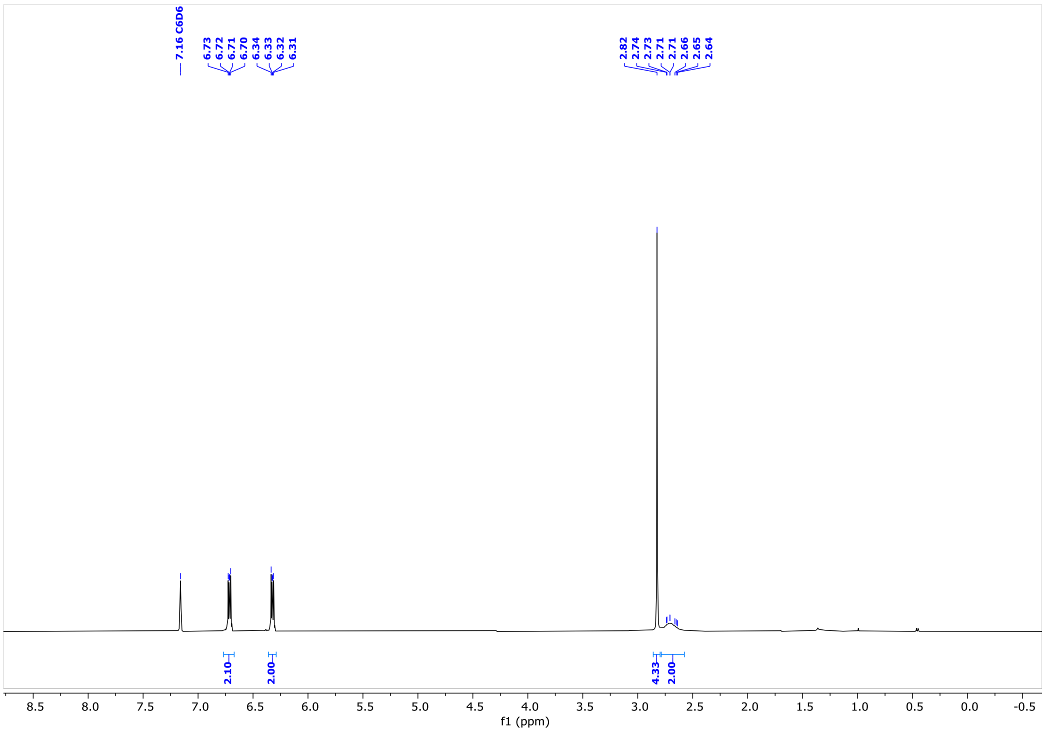

Supplementary Figure 25. ^{1}H NMR spectrum of **2i** in C_6D_6 at 300K.

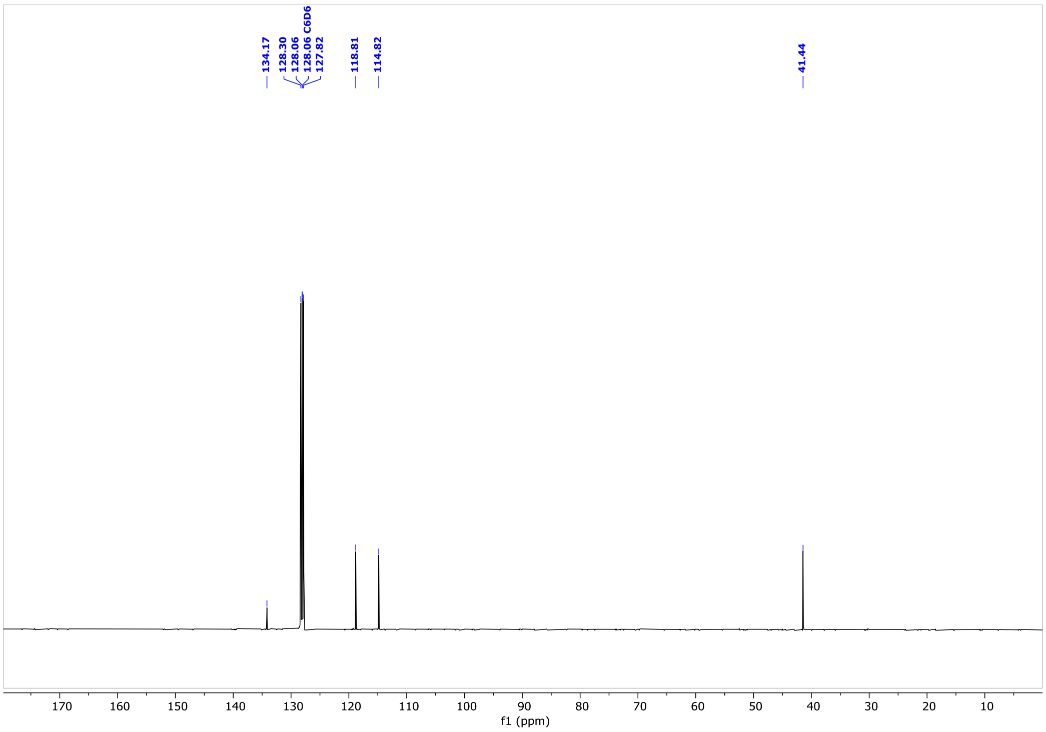

Supplementary Figure 26. ^{13}C{^{1}H} NMR spectrum of **2i** in C_6D_6 at 300K.

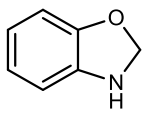

2,3-dihydrobenzo[*d*]oxazole (2j): ^{1}H NMR (400 MHz, C_6D_6) δ 6.91 (t, *J* = 7.9 Hz, 1H), 6.74 – 6.61 (m, 1H), 6.53 (dd, *J* = 7.7, 1.5 Hz, 1H), 6.40 (d, *J* = 7.7 Hz, 1H), 3.72 (s, 1H), 2.37 (s, 2H). ^{13}C NMR (101 MHz, C_6D_6) δ 144.01, 138.85, 121.90, 117.39, 114.33, 111.42, 30.42.

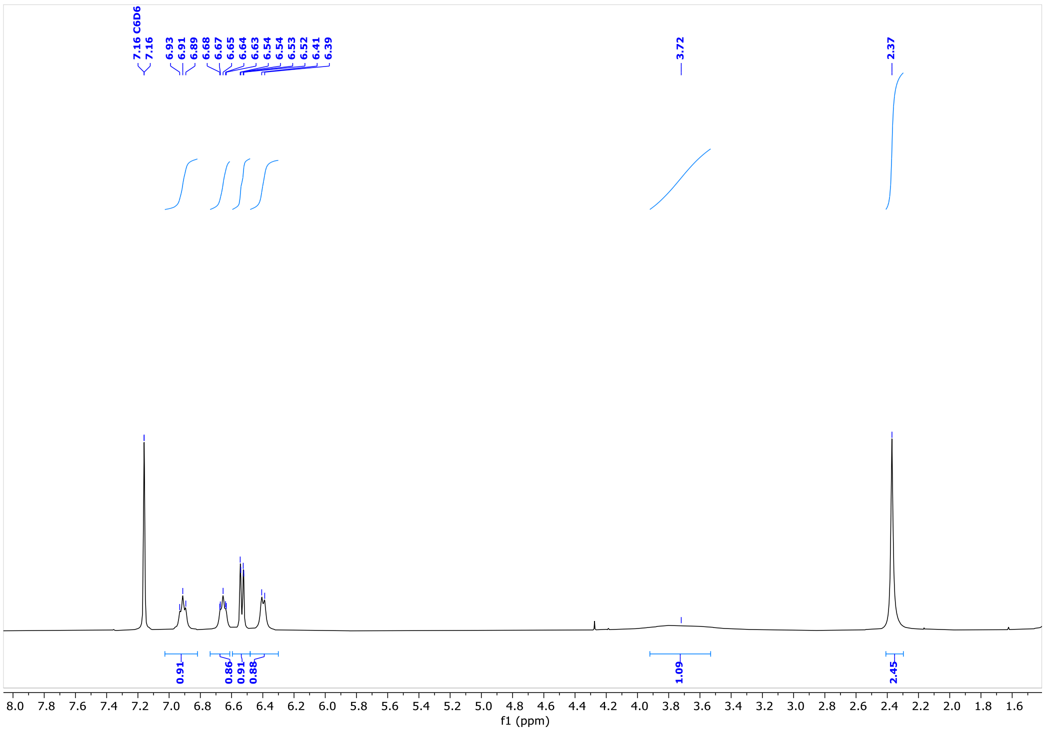

Supplementary Figure 27. ^{1}H NMR spectrum of **2j** in C_6D_6 at 300K.

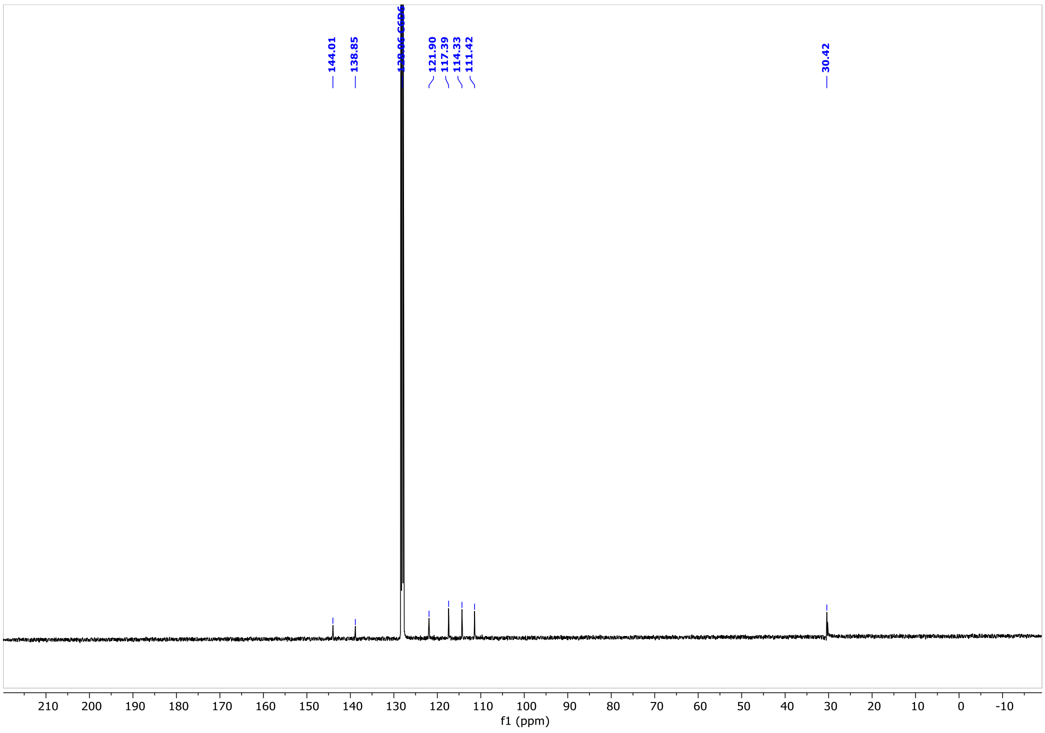

Supplementary Figure 28. ^{13}C{^{1}H} NMR spectrum of **2j** in C_6D_6 at 300K.

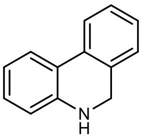

5,6-dihydrophenanthridine (2k): ^{1}H NMR (400 MHz, C_6D_6) δ 7.60 (dd, *J* = 7.8, 1.4 Hz, 1H), 7.57 – 7.50 (m, 1H), 7.10 (td, *J* = 7.6, 1.4 Hz, 1H), 7.06 – 6.99 (m, 2H), 6.85 – 6.75 (m, 2H), 6.29 (dd, *J* = 8.0, 1.2 Hz, 1H), 3.87 (s, 2H), 2.99 (s, 1H). ^{13}C NMR (101 MHz, C_6D_6) δ 146.42, 133.23, 132.85, 129.02, 127.14, 126.12, 123.96, 122.75, 122.22, 119.26, 115.30, 46.49.

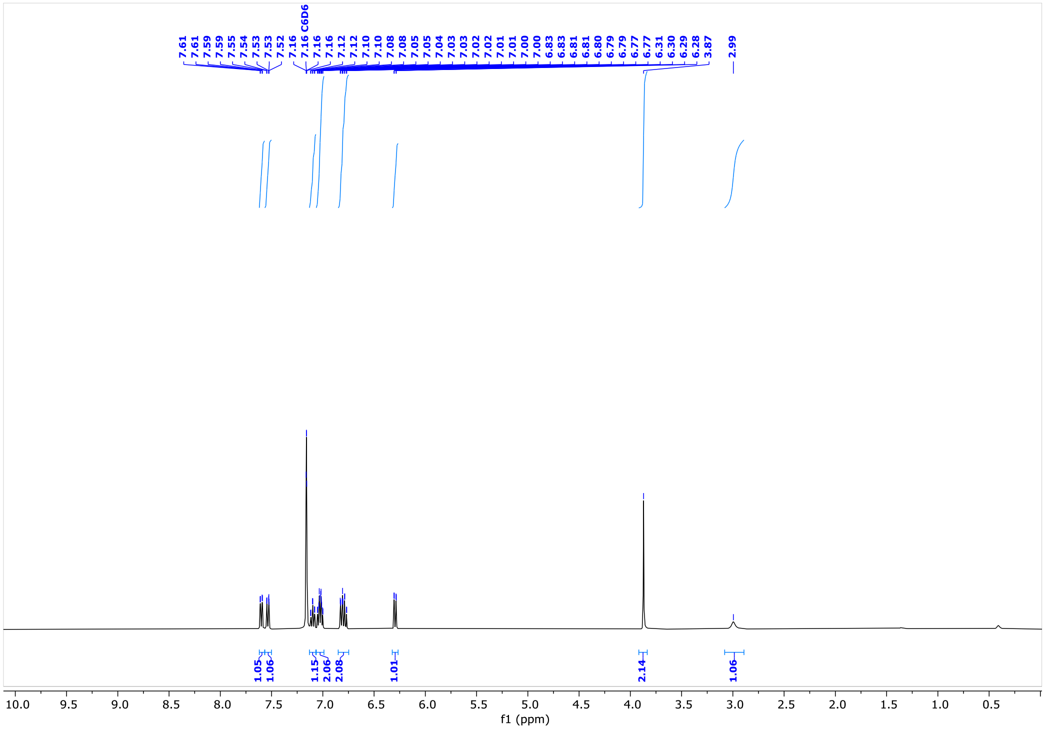

Supplementary Figure 29. ^{1}H NMR spectrum of **2k** in C_6D_6 at 300K.

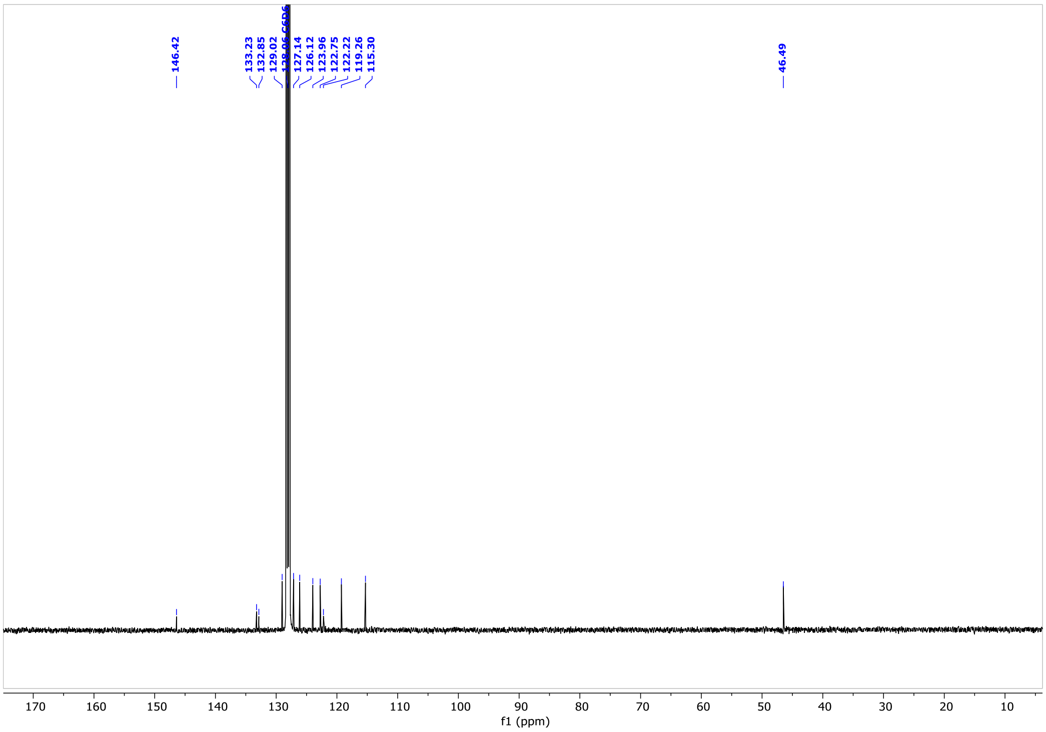

Supplementary Figure 30. ^{13}C{^{1}H} NMR spectrum of **2k** in C_6D_6 at 300K.

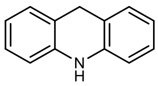

tetrahydroacridine (2l): ^{1}H NMR (400 MHz, C_6D_6) δ 7.01 (td, *J* = 7.4, 1.6 Hz, 2H), 6.95 – 6.87 (m, 2H), 6.82 (td, *J* = 7.4, 1.2 Hz, 2H), 6.24 (dd, *J* = 7.9, 1.2 Hz, 2H), 5.20 (s, 1H), 3.77 (s, 2H). ^{13}C NMR (101 MHz, C_6D_6) δ 140.57, 128.97, 127.13, 120.85, 120.29, 113.82, 31.58.

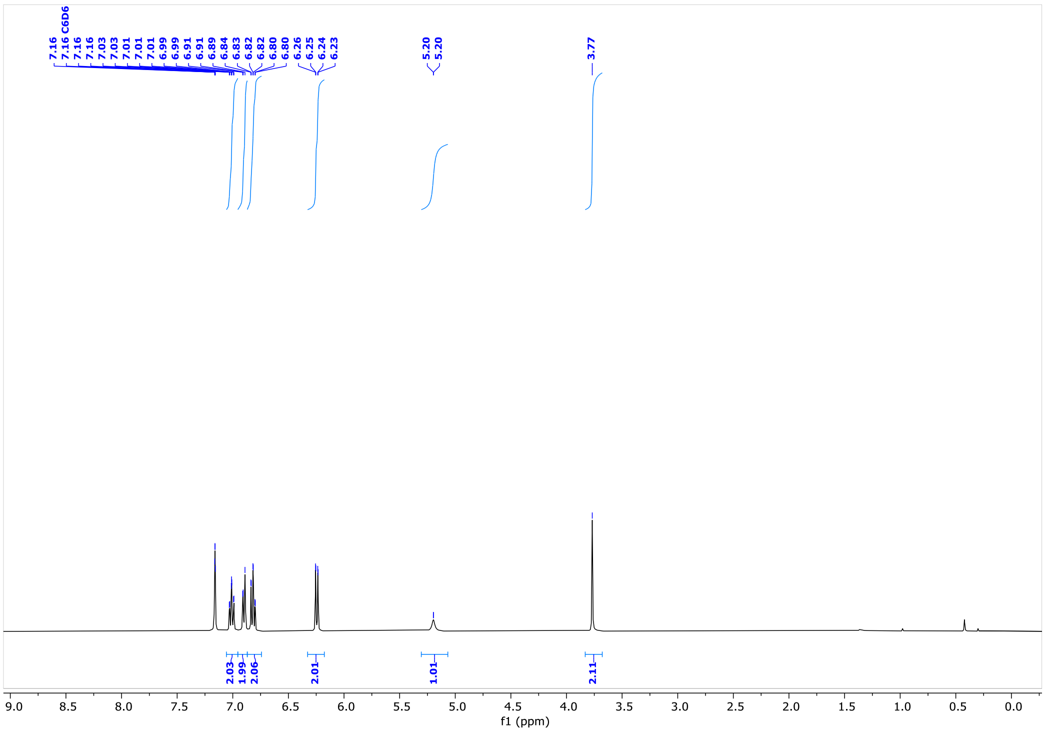

Supplementary Figure 31. ^{1}H NMR spectrum of **2l** in C_6D_6 at 300K.

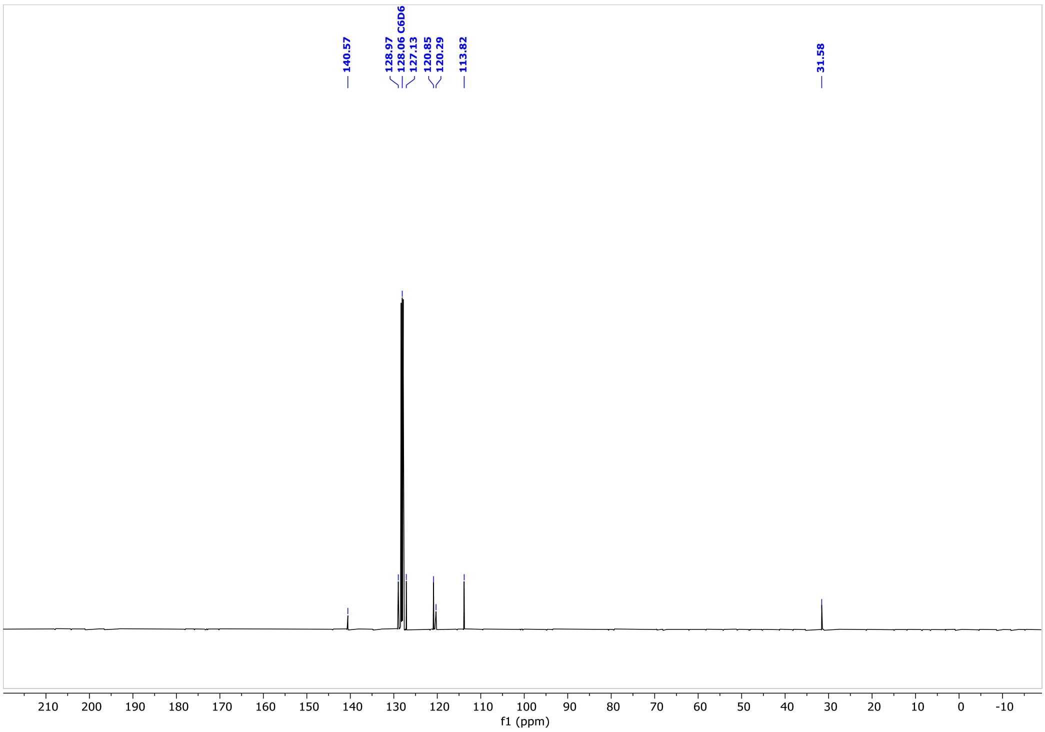

Supplementary Figure 32. ^{13}C{^{1}H} NMR spectrum of **2l** in C_6D_6 at 300K.

1.4 Mechanistic Studies

1.4.1 Competitive reaction between quinoline and H_3NBH_3

In glovebox, dialumene **Al-1** (10 mg), ammonia borane (0.43 mg), quinoline (1.77 mg or 3.54 mg) and C_6D_6 (0.3 mL) were mixed in a J-Young tube. The reaction mixture was monitored by ^1H NMR and aluminum hydride **Al-3** was detected as the major product.

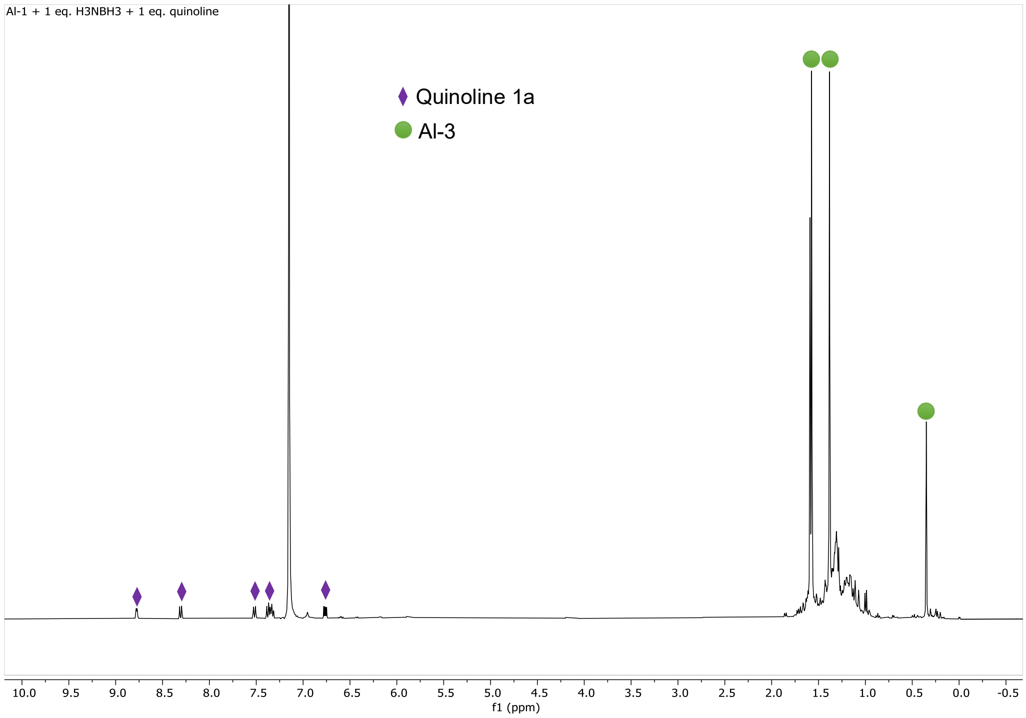

Supplementary Figure 33. Reaction of **Al-1** with 1 eq. H_3NBH_3 and 1 eq. quinoline.

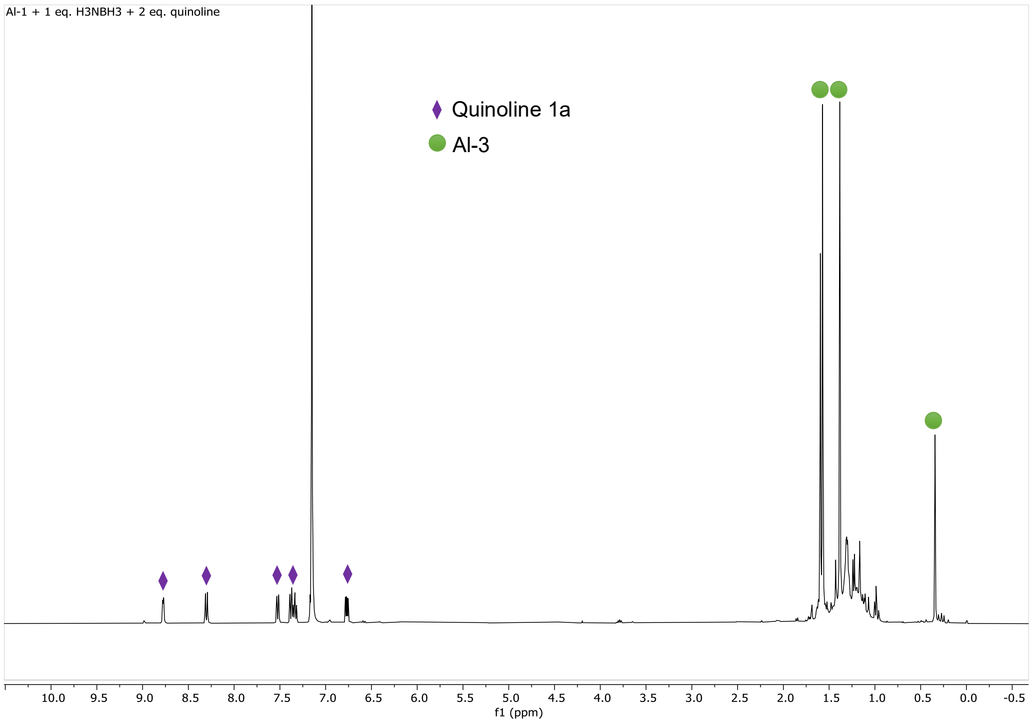

Supplementary Figure 34. Reaction of **Al-1** with 1 eq. H_3NBH_3 and 2 eq. quinoline.

1.4.2 Stoichiometric reactions of Al-2(or Al-3) with quinoline or H_3NBH_3

In glovebox, **Al-2** (or **Al-3**) (5 mg), ammonia borane and C_6D_6 (0.3 mL) were added sequentially to a J-Young tube. The reaction mixture was monitored by ^1H NMR.

The reaction between **Al-3** and quinoline indicated that **Al-3** is unreactive toward quinoline (Supplementary Fig. 35, Equation (1)). The reaction of **Al-3** with 2 eq. of ammonia borane and 3 eq. of quinoline formed 1,2-DHQ and hydrogenated NHC (Supplementary Fig. 35, Equation (2), Supplementary Fig. 36). The reaction of **Al-2** with excess ammonia borane formed hydrogenated NHC and ill-defined byproducts (Supplementary Fig. 35, Equation (3), Supplementary Fig. 37). The reaction of **Al-2** with 3 eq. of ammonia borane and 2 eq. of quinoline formed 1,2-DHQ and hydrogenated NHC (Supplementary Fig. 35, Equation (4), Supplementary Fig. 38). The reaction of **Al-3** with excess ammonia borane formed hydrogenated NHC and ill-defined byproducts (Supplementary Fig. 35, Equation (5), Supplementary Fig. 39).

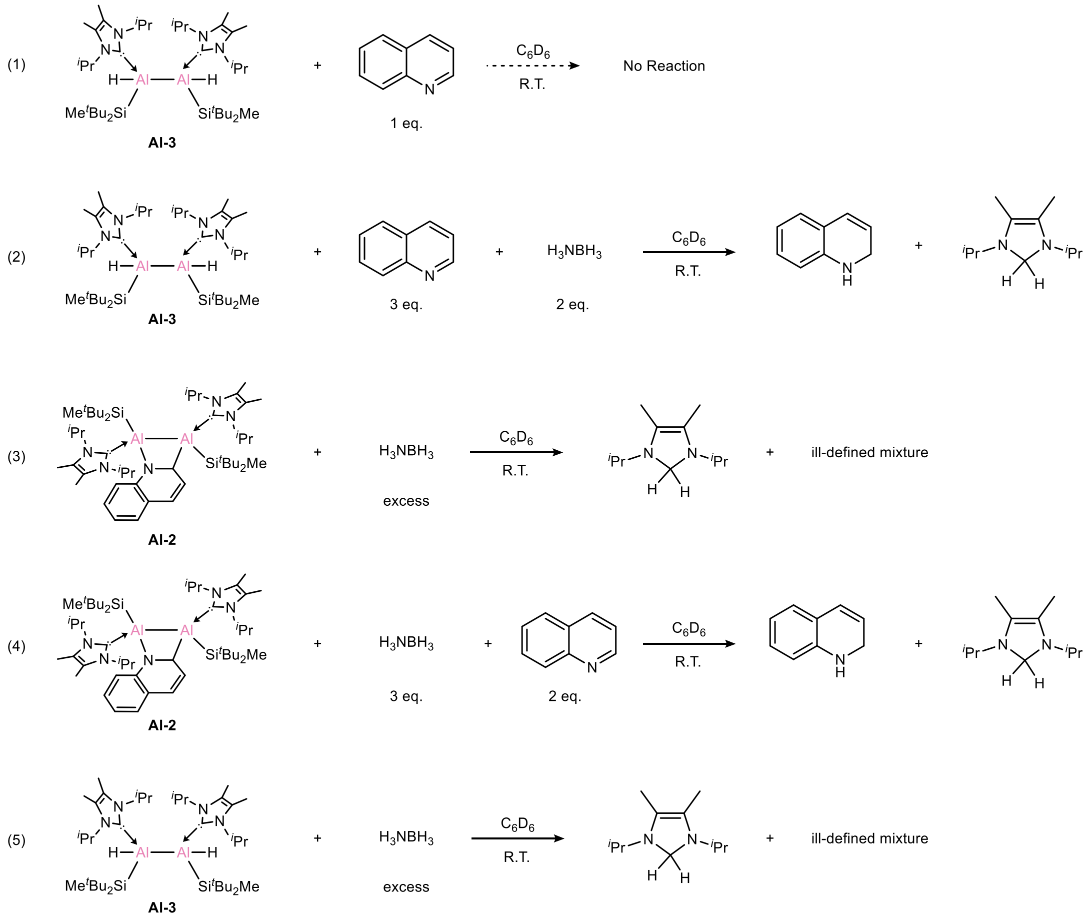

Supplementary Figure 35. Stoichiometric reactions of **Al-2**/**Al-3** with quinoline/H_3NBH_3.

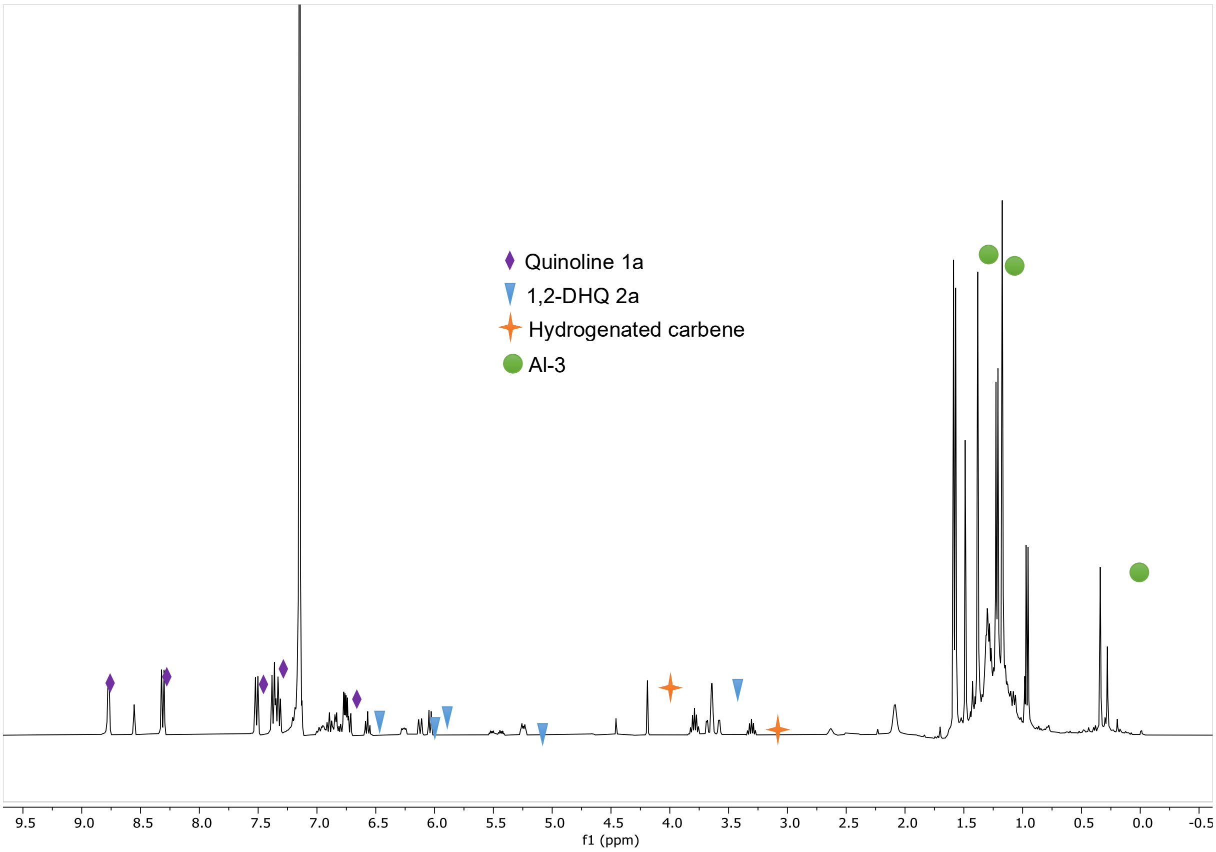

Supplementary Figure 36. ^{1}H NMR of the reaction of **Al-3** with 2 eq. H_3NBH_3 and 3 eq. quinoline.

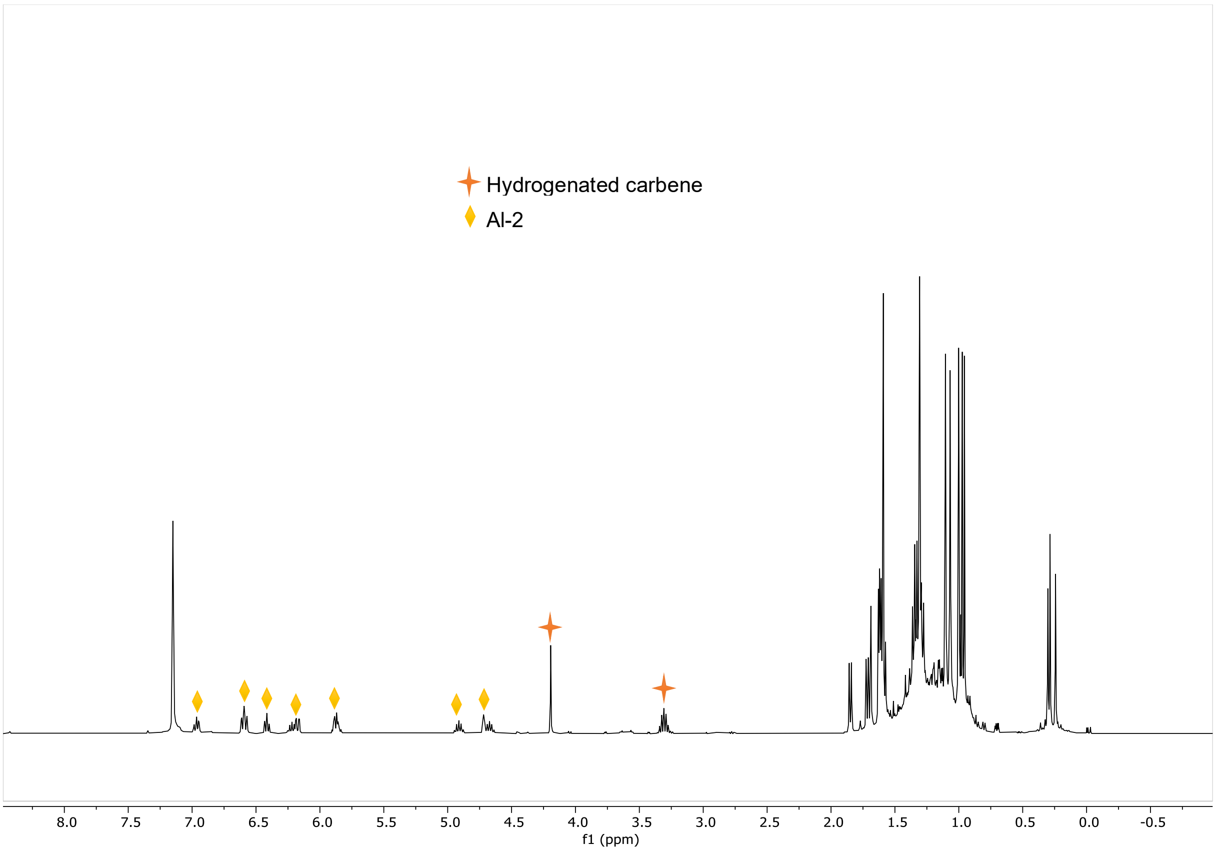

Supplementary Figure 37. ^{1}H NMR of the reaction of **Al-2** with H_3NBH_3.

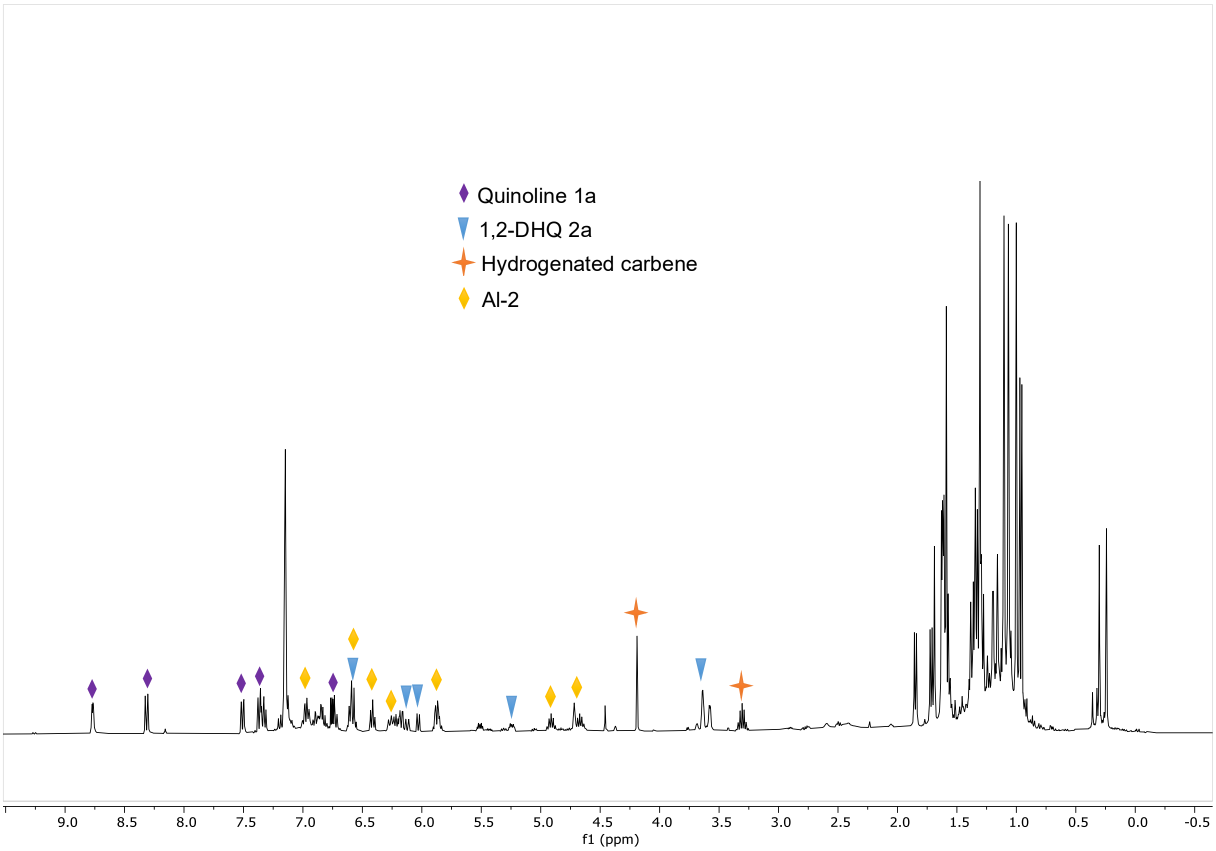

Supplementary Figure 38. ^{1}H NMR of the reaction of **Al-2** with 3 eq. H_3NBH_3 and 2 eq. quinoline.

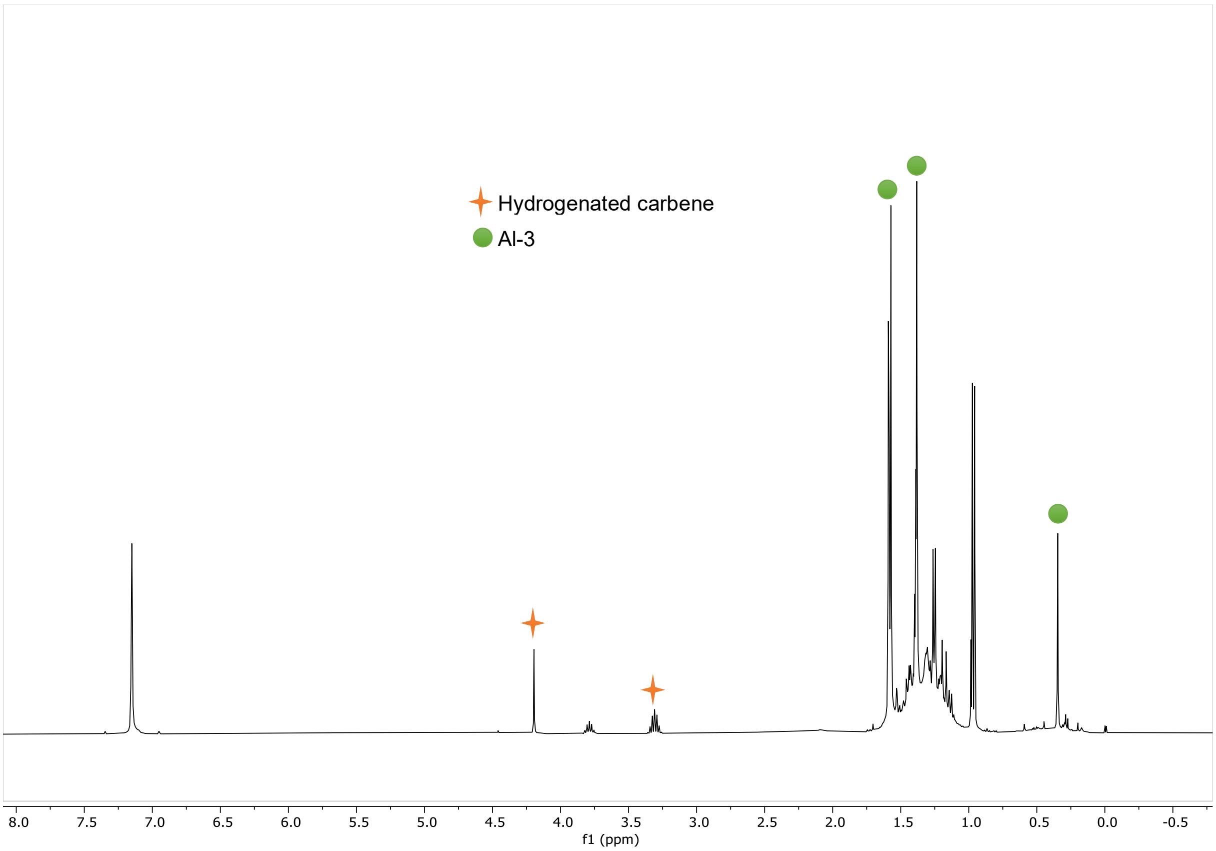

Supplementary Figure 39. ^{1}H NMR of the reaction of **Al-3** with H_3NBH_3.

In addition, hydrogenated NHC was independently synthesized by the reaction of free NHC with ammonia borane (Supplementary Fig. 40).

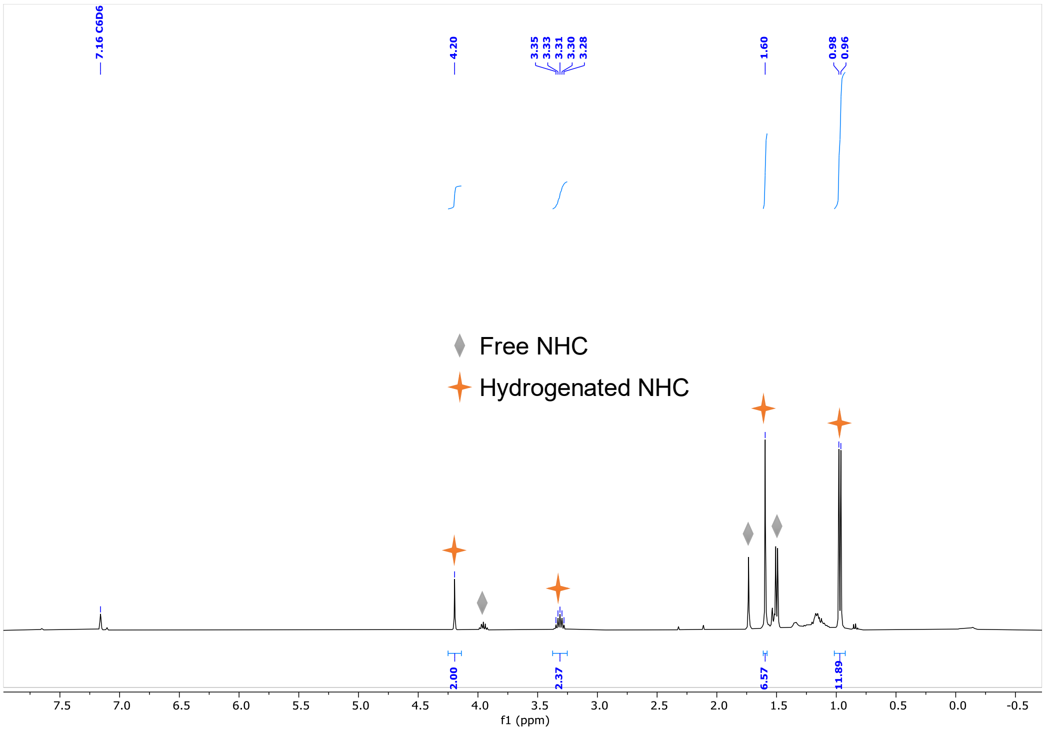

Supplementary Figure 40. ^{1}H NMR spectrum of hydrogenated NHC in C_6D_6 at 300K.

1.4.3 Deuterium-labelling experiments

In glovebox, dialumene **Al-1** (5 mol%), D_3NBH_3 (or H_3NBD_3) (0.1 mmol), quinoline substrate (0.1 mmol) and C_6D_6 (0.5 mL) were added sequentially to a 4 mL vial. The reaction mixture was stirred at room temperature and was monitored by ^{1}H NMR. After completion, the resulting solution was concentrated in vacuum and the residue was purified by silica gel column chromatography to give the corresponding **D-2a** or **D-2a'**, respectively.

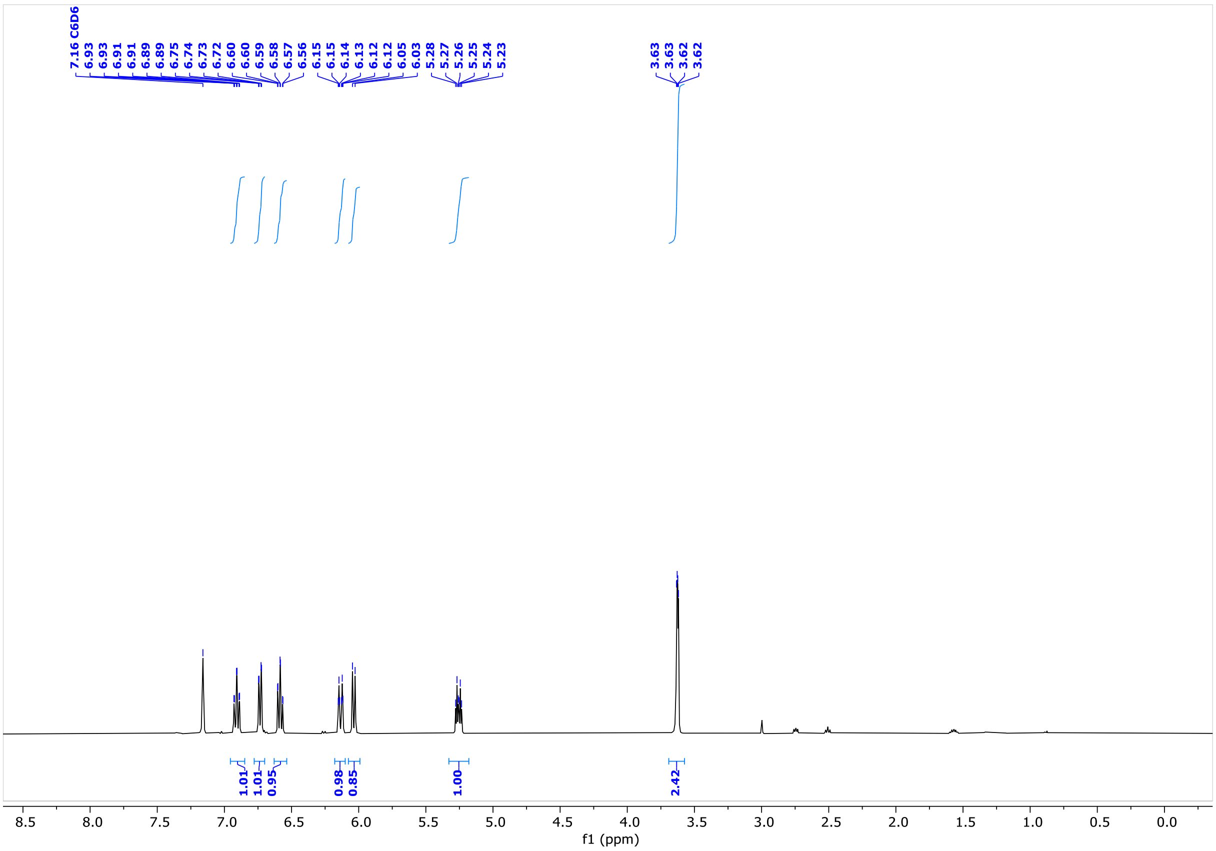

Supplementary Figure 41. ^{1}H NMR spectrum of **D-2a** in C_6D_6 at 300K.

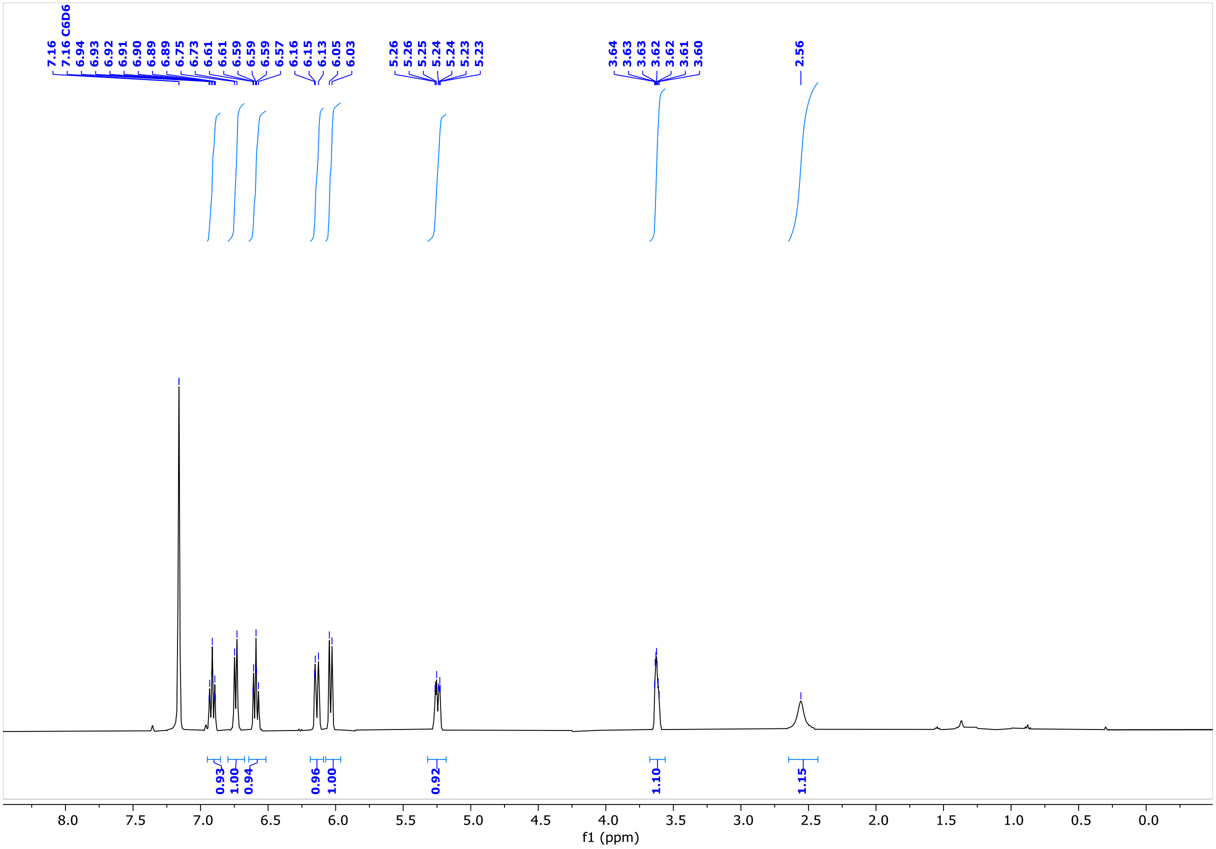

Supplementary Figure 42. ^{1}H NMR spectrum of **D-2a’** in C_6D_6 at 300K.

1.4.4 KIE experiments

The reaction was monitored by ^{1}H NMR, and data points before 20% conversion were subjected to the linear regression analysis.

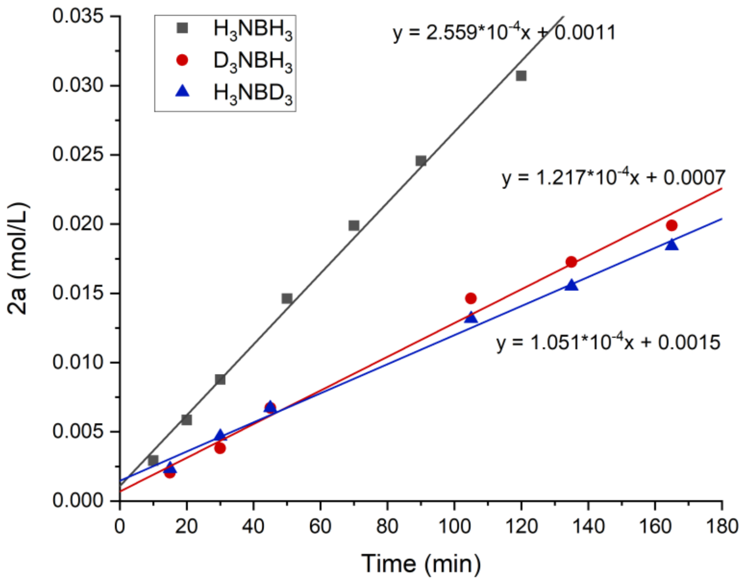

Supplementary Figure 43. KIE for catalytic reduction of **1a**.

1.4.5 Kinetic experiments

The reaction order toward quinoline **1a** was determined by varying the concentration of **1a** while keeping a constant concentration of both H_3NBH_3 and **Al-1** (Supplementary Fig. 44). The reaction was monitored by ^{1}H NMR at room temperature, and data points before 20% conversion were subjected to the linear regression analysis to determine the initial reaction rate.

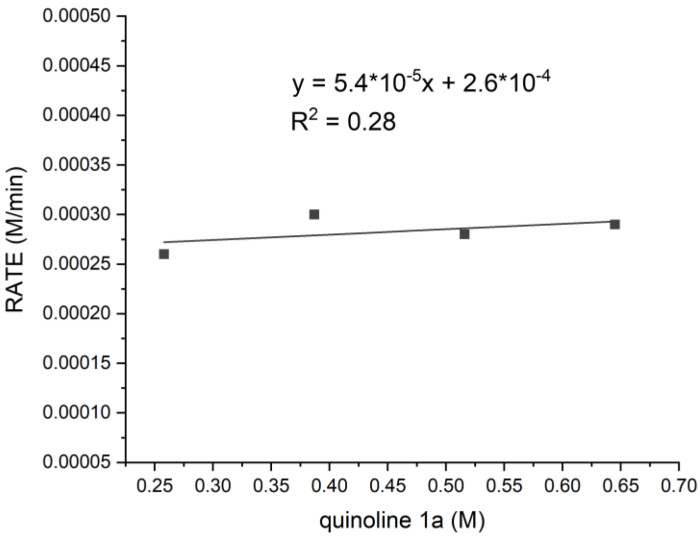

Supplementary Figure 44. Plot of [**1a**] vs. reaction rate.

The reaction order toward H_3NBH_3 was determined by varying the concentration of H_3NBH_3 while keeping a constant concentration of both **1a** and **Al-1** (Supplementary Fig. 45). The reaction was monitored by ^{1}H NMR at room temperature, and data points before 20% conversion were subjected to the linear regression analysis to determine the initial reaction rate.

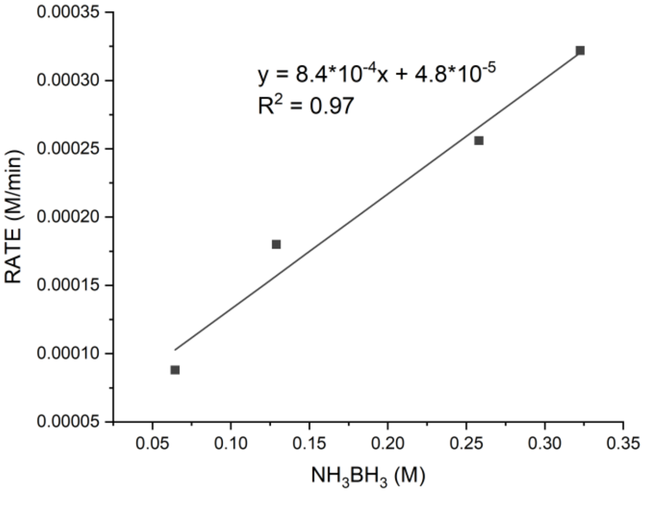

Supplementary Figure 45. Plot of [H_3NBH_3] vs. reaction rate.

The reaction order toward **Al-1** was determined by varying the concentration of **Al-1** while keeping a constant concentration of both **1a** and H_3NBH_3 (Supplementary Fig. 46). The reaction was monitored by ^{1}H NMR at room temperature, and data points before 20%

conversion were subjected to the linear regression analysis to determine the initial reaction rate.

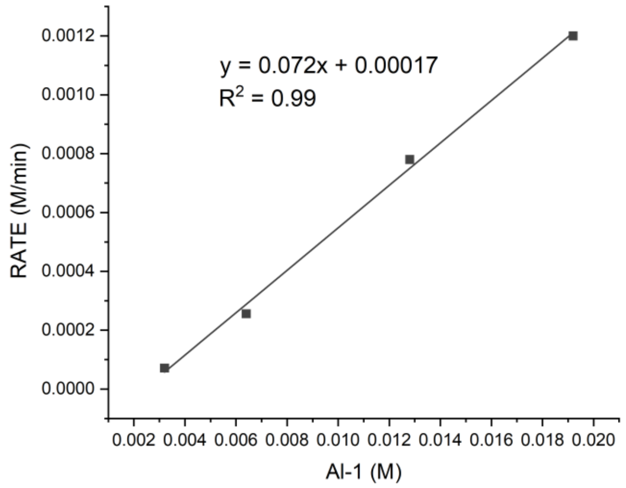

Supplementary Figure 46. Plot of [**Al-1**] vs. reaction rate.

2 Single Crystal X-Ray Structure Determination

Single crystal diffraction data were collected on a single-crystal X-ray diffractometer equipped with a Charge-Integrating Pixel Array Detector (Brucker Photon-II), a Microfocus X-Ray Source with a CuK_α ($\lambda = 1.54178$) or a Turbo X-Ray Source rotating anode with MoK_α radiation ($\lambda = 0.71073$ Å) and a Helios optic using the APEX4 software package[1] on single crystals coated with Fomblin®Y as perfluorinated ether. The single crystals were picked on a micro sampler, transferred to the diffractometer, and measured frozen under a stream of cold nitrogen (100 K). A matrix scan was used to determine the initial lattice parameters. Reflections were merged and corrected for Lorenz and polarization effects, scan speed, and background using SAINT.[2] Absorption corrections, including odd and even ordered spherical harmonics were performed using SADABS.[2] Space group assignments were based upon systematic absences, E statistics, and successful refinement of the structures. Structures were solved by direct methods with the aid of successive difference Fourier maps and were refined against all data using the APEX IV software in conjunction with SHELXL-2014[3] and SHELXLE.[4] H atoms were placed in calculated positions and refined using a riding model, with methylene and aromatic C–H distances of 0.99 and 0.95 Å, respectively, and Uiso(H) = 1.2·Ueq(C). Non-hydrogen atoms were refined with anisotropic displacement parameters. Full-matrix least-squares refinements were carried out by minimizing Σw(Fo2-Fc2)2 with the SHELXL weighting scheme.[5] Neutral atom scattering factors for all atoms and anomalous dispersion corrections for the non-hydrogen atoms were taken from International Tables for Crystallography.[6] The images of the crystal structures were generated by Mercury.[7] The CCDC numbers 2441311-2441314 contain the supplementary crystallographic data for the structures **Al-2**, **Al-3**, **Al-4** and **2b**. These data can be obtained free of charge from the Cambridge Crystallographic Data Centre via https://www.ccdc.cam.ac.uk/structures/.

Supplementary Table 4 Crystallographic details

	**Al-2**	**Al-3**
**CCDC-Number**	2441311	2441312
Chemical formula	$C_{49}H_{89}Al_2N_5Si_2$, 1.5(C_6H_6)	$C_{40}H_{84}Al_2N_4Si_2$
M_r	975.55	731.25
Crystal system, space group	Monoclinic, *P* 1 21/n 1	Orthorhombic, *P* 21 21 2
Temperature (K)	100	100
a (Å), α(°)<br>*b (Å)*, β(°)<br>*c (Å)*, γ(°)	11.5068(7), 90<br>33.620(2), 101.774(3)<br>15.7483(11), 90	12.6898(9), 90<br>17.9576(11), 90<br>11.5027(9), 90
V (Å^3)	5964.2(7)	2621.2(3)
Z	4	2
F(000)	2140	812
D_x (g/cm^3)	1.086	0.926
Radiation type	Mo *K*α	Mo *K*α
μ (mm^{-1})	0.128	0.127
θ range (°) for cell meas.	2.35–26.33	2.39–25.64
Crystal size (mm)	0.236 × 0.159 × 0.105	0.324 × 0.22 × 0.216
Diffractometer	Bruker Photon CMOS	Bruker Photon CMOS
Radiation source	IMS rotating anode	TXS rotating anode
Monochromator	Helios optic	Helios optic
Absorption correction	Multi-scan	Multi-scan
T_{min}, T_{max}	0.4603, 0.7454	0.6303, 0.7543
θ_{max} (°)	25.681	25.926
Range of *h*, *k*, *l*	*h* = -14→14, *k* = -41→41, *l* = -18→19	*h* = -15→15, *k* = -21→21, *l* = -14→13
Refinement method	Full-matrix least-squares on F^2	Full-matrix least-squares on F^2
Data/restraints/parameters	11311/0/630	5073/375/357
Goodness-of-fit on F^2	1.089	1.036
Final *R* indices (I>2σ(I))	R_1 = 0.0569, wR_2 = 0.1382	R_1 = 0.0449, wR_2 = 0.1343
$\Delta\rho_{max}$, $\Delta\rho_{min}$ (e Å^{-3})	0.366, -0.406	0.170, -0.156

	**Al-4**	**2b**
**CCDC-Number**	2441313	2441314
Chemical formula	$C_{45}H_{87}Al_2N_5Si_2$	$C_9H_7F_2N$
M_r	808.33	167.16
Crystal system, space group	Monoclinic, *P* 1 n 1	Orthorhombic, *P* b c a
Temperature (K)	100	100
a (Å), α(°)<br>*b (Å)*, β(°)<br>*c (Å)*, γ(°)	13.2978(8), 90<br>11.4015(6), 106.403(2)<br>19.3025(12), 90	17.251(3), 90<br>5.0448(9), 90<br>17.406(3), 90
V (Å^3)	2807.4(3)	1514.9(5)
Z	2	8
F(000)	892	688
D_x (g/cm^3)	0.956	1.466
Radiation type	Mo *K*α	Mo *K*α
μ (mm^{-1})	0.125	0.122
θ range (°) for cell meas.	2.44–25.66	2.34–25.63
Crystal size (mm)	0.216 × 0.189 × 0.132	0.378 × 0.255 × 0.176
Diffractometer	Bruker Photon CMOS	Bruker Photon CMOS
Radiation source	TXS rotating anode	TXS rotating anode
Monochromator	Helios optic	Helios optic
Absorption correction	Multi-scan	Multi-scan
T_{min}, T_{max}	0.7168, 0.7453	0.6028, 0.7453
θ_{max} (°)	25.798	25.655
Range of *h*, *k*, *l*	*h* = -16→16, *k* = -13→13, *l* = -23→23	*h* = -20→20, *k* = -6→6, *l* = -21→21
Refinement method	Full-matrix least-squares on F^2	Full-matrix least-squares on F^2
Data/restraints/parameters	10684/86/513	1430/0/113
Goodness-of-fit on F^2	1.044	1.048
Final *R* indices (I>2σ(I))	R_1 = 0.0425, wR_2 = 0.1167	R_1 = 0.0363, wR_2 = 0.1018
$\Delta\rho_{max}$, $\Delta\rho_{min}$ (e Å^{-3})	0.478, -0.254	0.192, -0.263

ORTEP-style illustrations

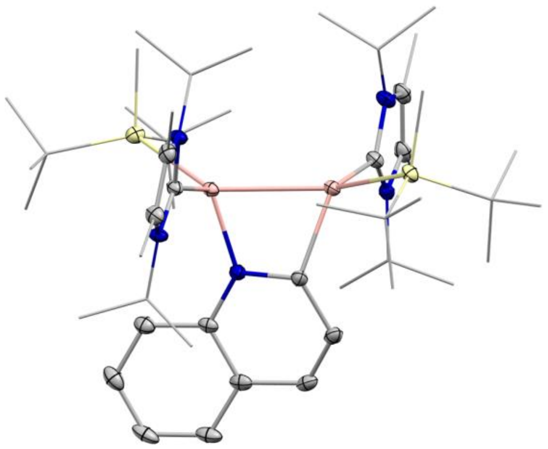

Supplementary Figure 47. Thermal ellipsoid plot (50% probability) of X-ray structure of compound **Al-2**.
CCDC: 2441311.

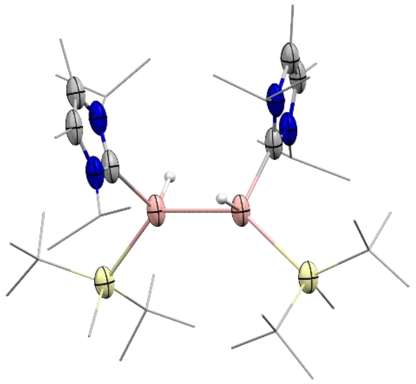

Supplementary Figure 48. Thermal ellipsoid plot (30% probability) of X-ray structure of compound **Al-3**.
CCDC: 2441312.

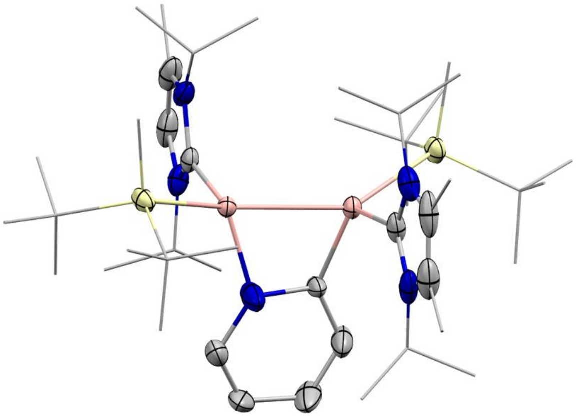

Supplementary Figure 49. Thermal ellipsoid plot (50% probability) of X-ray structure of compound **Al-4**.
CCDC: 2441313.

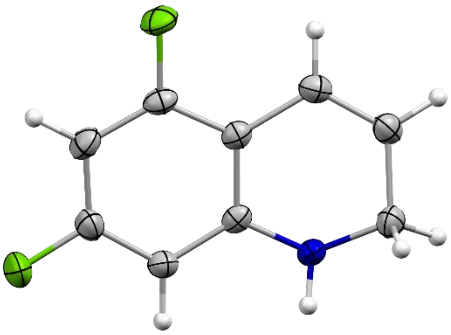

Supplementary Figure 50. Thermal ellipsoid plot (50% probability) of X-ray structure of compound **2b**.
CCDC: 2441314.

3 Computational Details

The quantum chemical calculations were conducted using ORCA 6 software.[8] Geometry optimizations were carried using the r^2SCAN-3c composite method,[9] utilizing the regularized and restored SCAN functional,[10-11] geometrical counterpoise correction gCP,[12] the atom-pairwise dispersion correction based on tight binding partial charges (D4),[13–15] the def2-mTZVPP basis set and def2-mTZVPP/J auxiliary basis set.[9] The optimized geometries were verified as minima or transition states by analytical frequency calculations. The transition states were additionally verified by IRC calculations. Single point calculations of the optimized geometries were conducted at the r^2SCAN-3c level using the SMD solvation module,[16] to obtain electrostatic contribution (Genp) and the cavity term (Gcds), in order to account for the solvent effects. For more accurate electronic energies, single point calculations of the optimized geometries were carried using the PW6B95[17] functional with D4 dispersion correction, def2-QZVPP[18] basis set, def2/J[19] and def2-QZVPP/C[20] auxiliary basis sets. The method at which the free energies were obtained is denoted as (SMD=Benzene)PWPB95-D4/def2-QZVPP//r^2SCAN-3c and the summary of the thermochemistry results is presented in Supplementary Table 5.

Supplementary Table 5. Calculated energies (E_h). Thermochemistry at 298.15 K. E_{PW6B95} - electronic energy at the PW6B95-D4/def2-QZVPP//r^2SCAN-3c level; G-E_{el} - Gibbs energy minus the electronic energy at the r^2SCAN-3c//r^2SCAN-3c level; G_{cds} - cavity term and G_{enp} - electrostatic contribution at the r^2SCAN-3c(SMD=Beneze)//r^2SCAN-3c level; G_{conc} - concentration-induced free-energy shift (G_{conc} = $\mathrm{RT}\ln(24.5)$); G_{PW6B95} - free energy at the (SMD=Benzene)PW6B95-D4/def2-QZVPP/ //r^2SCAN-3c level (G_{PW6B95} = E_{PW6B95} + [G-E_{el}] + G_{cds} + G_{enp} + G_{conc}).

Compound	E_{PW6B95}	G-E_{el}(298.15 K)	G_{cds}(Benzene)	G_{enp}(Benzene)	G_{conc}(298.15 K)	G_{PW6B95}
**Al-1**	-2860.82703	1.06704	-0.02025	-0.01804	0.00302	-2859.79527
**Al-2**	-3263.54069	1.19901	-0.02164	-0.02102	0.00302	-3262.38132
**Al-3**	-2862.07003	1.08433	-0.02022	-0.01727	0.00302	-2861.02017
**Al-3$^{X\text{-}ray}$**	-2862.06289	1.08438	-0.02376	-0.01756	0.00302	-2861.01681
**TS1**	-3263.45879	1.19773	-0.02762	-0.02149	0.00302	-3262.30716
**TS2**	-2944.19958	1.13070	-0.02267	-0.01625	0.00302	-2943.10479
**A**	-2986.70603	0.90653	-0.02091	-0.02318	0.00302	-2985.84057
**TS(A-B)**	-2986.67226	0.90312	-0.02096	-0.02465	0.00302	-2985.81173
**B**	-2986.70594	0.90853	-0.02053	-0.02536	0.00302	-2985.84028
**B'**	-2986.69093	0.90629	-0.02368	-0.02529	0.00302	-2985.83059
**TS(B'-C)**	-2986.67175	0.90478	-0.02300	-0.02539	0.00302	-2985.81233
**C**	-2986.71269	0.91025	-0.02378	-0.02540	0.00302	-2985.84861
**D**	-3070.08988	0.97918	-0.02510	-0.02160	0.00302	-3069.15438
**TS(D-E)**	-3070.07721	0.97552	-0.02283	-0.02170	0.00302	-3069.14320
**E**	-3070.07914	0.97752	-0.02266	-0.02160	0.00302	-3069.14287
**TS(E-F)**	-3070.07358	0.97685	-0.02386	-0.02160	0.00302	-3069.13917
**F**	-2987.88110	0.93004	-0.02251	-0.02315	0.00302	-2986.99369
**G**	-2584.02793	0.77718	-0.01875	-0.01872	0.00302	-2583.28520
**A $_{pyridine}$**	-2832.75840	0.86057	-0.01927	-0.02269	0.00302	-2831.93676

**TS(A-B)** $_{pyridine}$	-2832.73684	0.85883	-0.01958	-0.02296	0.00302	-2831.91754
**B** $_{pyridine}$	-2832.77771	0.86333	-0.01975	-0.02392	0.00302	-2831.95503
**B'** $_{pyridine}$	-2832.77508	0.86189	-0.02230	-0.02266	0.00302	-2831.95513
**TS(B'-C)** $_{pyridine}$	-2832.73838	0.86131	-0.02153	-0.02283	0.00302	-2831.91841
**C** $_{pyridine}$	-2832.77613	0.86549	-0.01925	-0.02302	0.00302	-2831.94988
**D** $_{pyridine}$	-2916.14300	0.93273	-0.02455	-0.02174	0.00302	-2915.25354
**TS(D-E)**$_{pyridine}$	-2916.13217	0.92892	-0.02233	-0.02133	0.00302	-2915.24389
**E** $_{pyridine}$	-2916.13460	0.93100	-0.02194	-0.02118	0.00302	-2915.24370
**TS(E-F)**$_{pyridine}$	-2916.12889	0.93088	-0.02258	-0.02130	0.00302	-2915.23888
**F** $_{pyridine}$	-2833.93737	0.88380	-0.02138	-0.02277	0.00302	-2833.09470
**G** $_{pyridine}$	-2430.08654	0.73143	-0.01606	-0.01741	0.00302	-2429.38555
**H_3NBH_3**	-83.36039	0.04633	-0.01468	0.00183	0.00302	-83.32389
**CTB**	-246.62155	0.12878	-0.01610	0.00286	0.00302	-246.50299
**IiPr**	-541.64311	0.25273	-0.01024	-0.01102	0.00302	-541.40862
**IiPrH_2**	-542.84976	0.27575	-0.00617	-0.00725	0.00302	-542.58441
**QNL**	-402.64116	0.10367	-0.00661	-0.00716	0.00302	-402.54825
**QNLH_2**	-403.83131	0.12563	-0.00772	-0.00752	0.00302	-403.71790

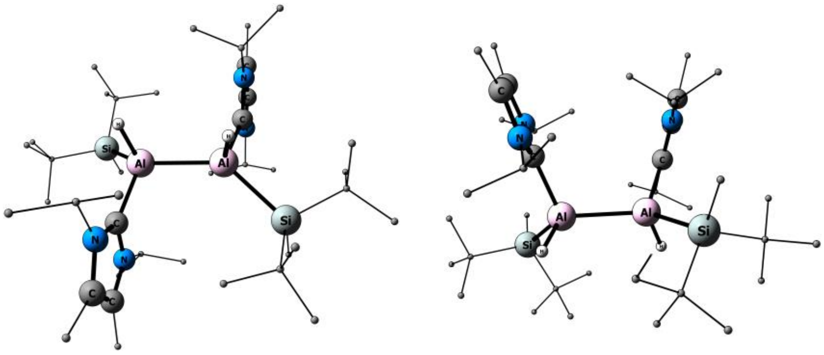

Supplementary Figure 51. Left – optimized structure of **Al-3** obtained from **TS2**; θ(H-Al-Al-H) = -19.6°, θ(Si-Al-Ai-Si) = 109.4°, θ(C-Al-Al-C) = -163.5°. Right – optimized X-ray structure of **Al-3$^{X\text{-}ray}$**; θ(H-Al-Al-H) = -92.4°, θ(Si-Al-Ai-Si) = 138.7°, θ(C-Al-Al-C) = 48.3°.

We propose that the active catalytic species is formed in the reaction via the pathway described in Supplementary Fig. 52. First, dialumene **Al-1** reacts with the ammonia borane complex to form the **Al-3**. This process is expected to proceed faster than the formation of **Al-2**, as described in the main text. Next one of the NHC ligands is replaced by a quinoline forming intermediate **I** via intermediate **H**. At this stage the free carbene is hydrogenated by an additional equivalent of the ammonia borane complex. **I** converts to intermediate **J**, achieving the addition of the quinoline across the Al-Al bond, similarly to the formation of **Al-2**. Compound **J** rearranges to **K** in which there is a coordination site for an additional quinoline molecule. Upon coordination intermediate **L** is formed. **L** can undergo the NHC – quinolone exchange accompanied by NHC hydrogenation. Thus, first NHC dissociates forming intermediate **M**, which upon binding to a quinoline forms the proposed catalytic species **A**. The free carbene is then hydrogenated in the presence of the ammonia borane complex.

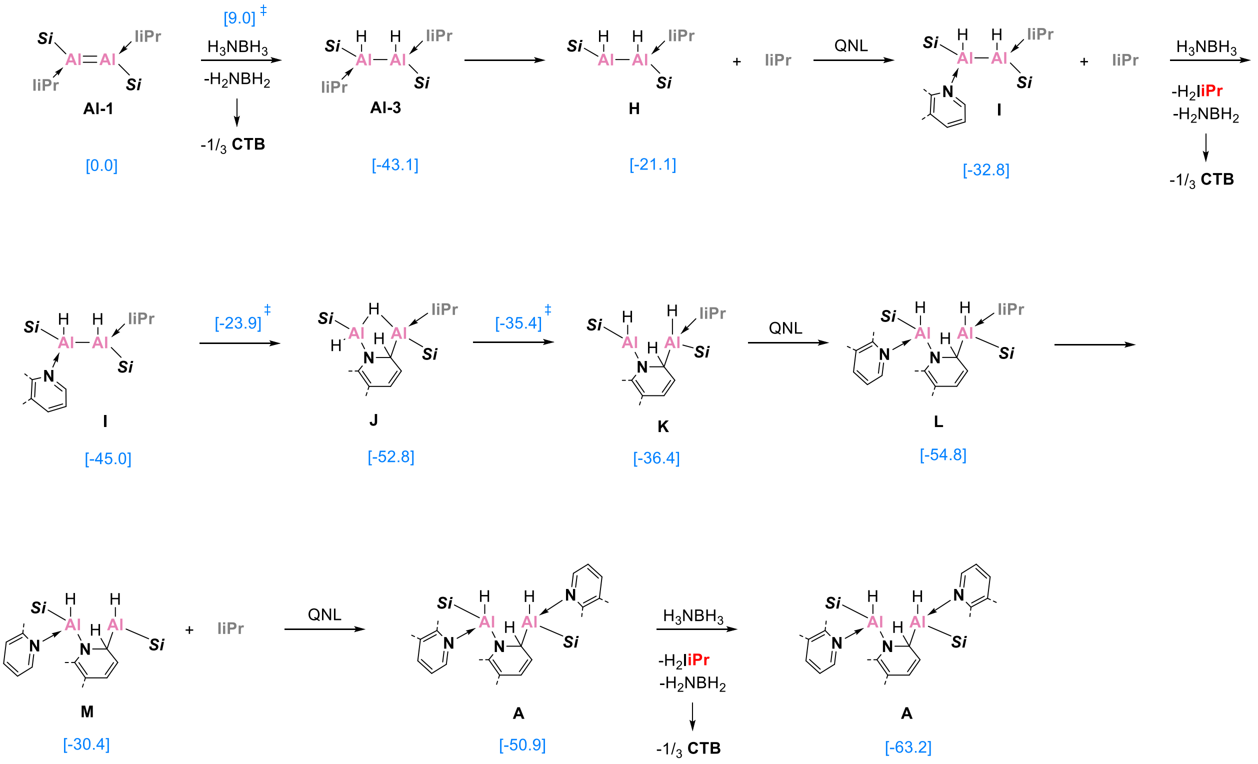

Supplementary Figure 52. The proposed mechanism of the formation of the catalytic species **A**. The free energies (at (SMD=Benzene)PW6B95-D4/def2-QZVPP//r^{2}SCAN-3c level of theory) relative to the starting compound **Al-1** are shown in brackets.

To demonstrate the non-covalent interaction between the quinoline moieties, we use the reduced density gradient (RDG) function. The isosurface (iso = 0.5) represents the weak interaction regions between aromatic moieties (Supplementary Fig. 53).

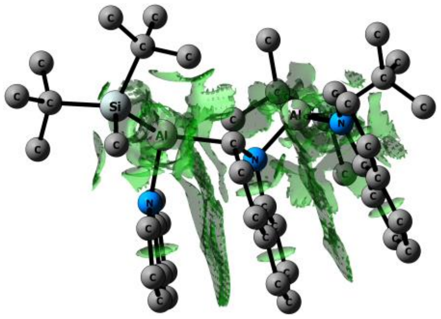

Supplementary Figure 53. Isosurface of the reduced density gradient (RDG) function of **A**, at PBE0-D4/def2-TZVP//r^{2}SCAN-3c level of theory.

The quantum theory of atom in molecules (QTAIM) molecular graph (Supplementary Fig. 54) also displays interactions between the quinoline moieties, showing seven (3, −1) critical points. These calculations along with the geometrical features support the assignment of the existing π-π stacking interactions between the quinoline moieties in **A.**

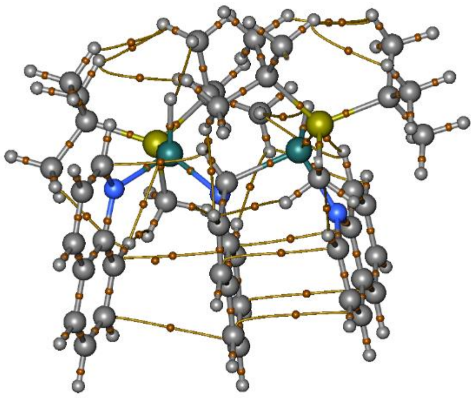

Supplementary Figure 54. QTAIM molecular graph of **A** showing the bond paths and bond critical points, at PBE0-D4/def2-TZVP//r^{2}SCAN-3c level of theory.

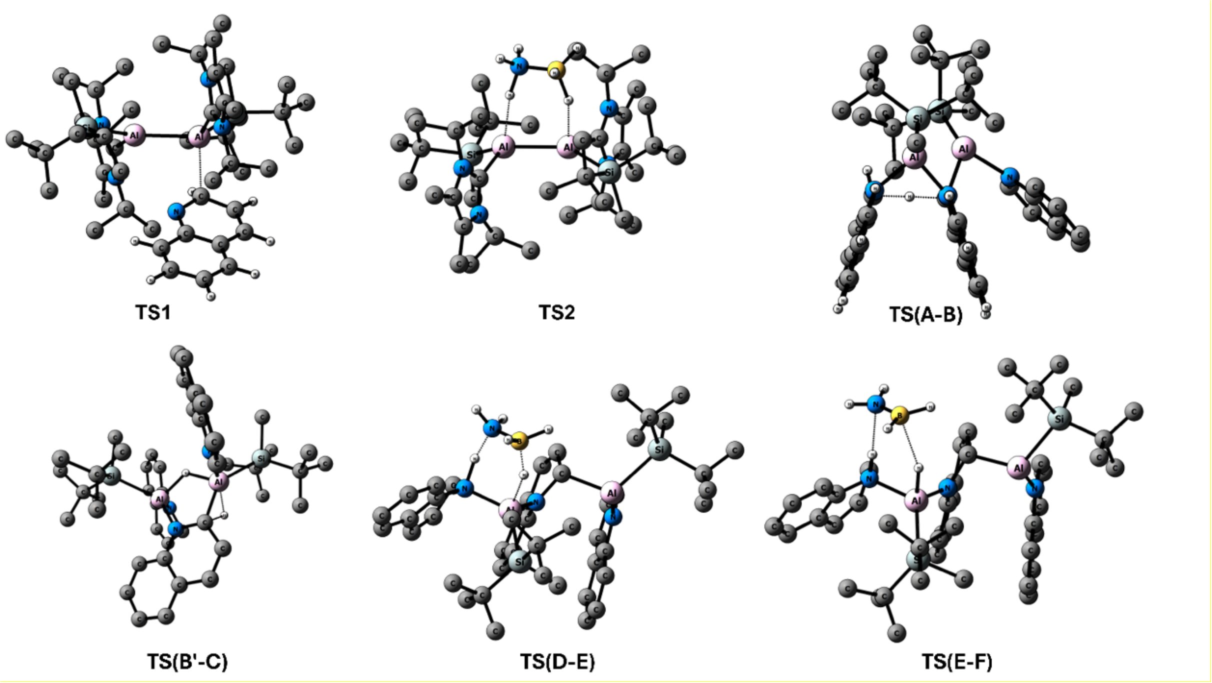

Supplementary Figure 55. Transition states for the proposed mechanism of the catalytic 1,2-reduction of quinoline.

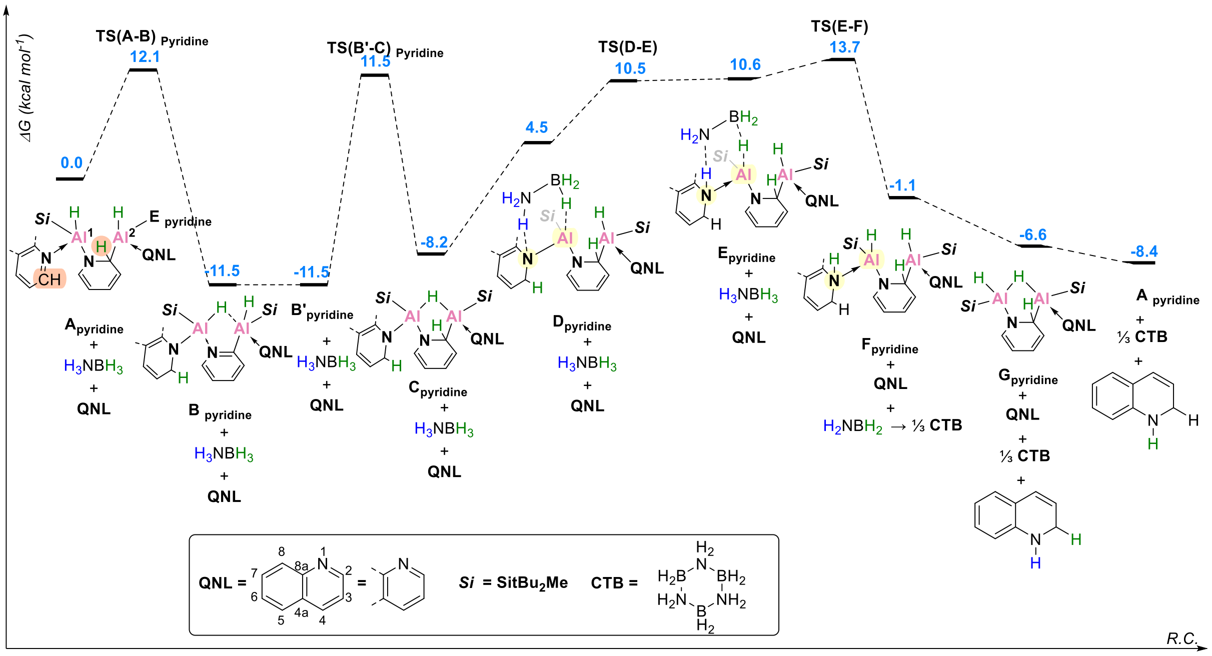

Supplementary Figure 56. Calculated reaction pathway for the proposed mechanism of the catalytic 1,2-reduction of quinoline by the pyridine-bridged complex $\mathbf{A}_{\mathbf{pyridine}}$. Free energies at the (SMD=Benzene)PW6B95-D4/def2-QZVPP//r2SCAN-3c level of theory.

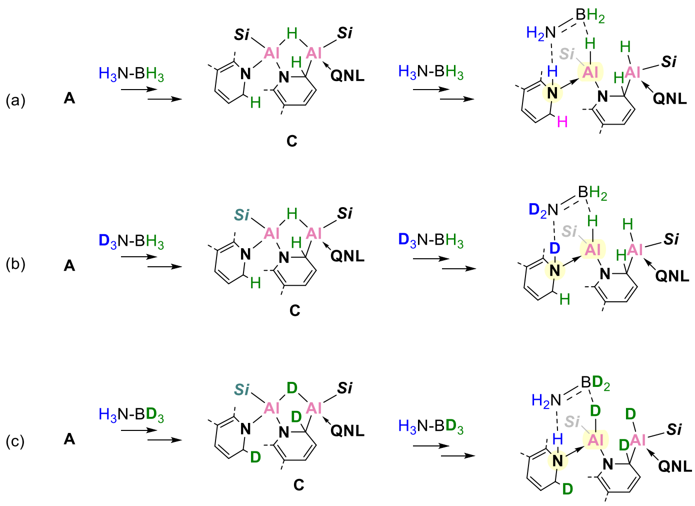

Supplementary Figure 57. The proposed isotopies for the rate determining barrier **C→TS(E-F)**.

According to the proposed mechanism in the process of the catalytic reaction different isotopomers of the reactive intermediate **C** and the rate determining transition state **TS(E-F)** are expected to form if the reaction is carried out in unlabeled or partially labeled ammonia borane (Supplementary Fig. 57). The free energy difference between the resulting isotopomers of **C** and isotopomers of **TS(E-F)** can rationalize the experimentally observed kinetic isotope effect. In the case of H_3NBH_3 and D_3NBH_3 similar intermediate **C** is expected to form (Supplementary Fig. 57, reaction (a) and (b)). These reactions would defer in **TS(E-F)**, where in the case of D_3NBH_3 the deuterium is expected to be transferred to the quinoline. When using H_3NBD_3, the deuterated intermediate **C** is expected to form in the course of the reaction (Supplementary Fig. 57, reaction (c)). Additionally, in the corresponding **TS(E-F)** a deuterium atom will be transferred from the boron atom to Al[1]. Calculations show that the ΔΔG(**C-TS(E-**

F)) for reactions (b) and (c) relatively to reaction (a) are 0.16 kcal mol^{-1} and 0.35 kcal mol^{-1}, respectively.

Calculations show that the presence of a methyl substituent of the C2 position is expected to have a dramatic effect kinetics of the reaction of the respective quinoline with the dialumene. The barrier is higher by 12.7 kcal mol^{-1}. With $\Delta G^{\ddagger}$ = 35.5 kcal mol^{-1} the reaction with 2-methylquinoline is not expected to occur (Supplementary Fig. 58).

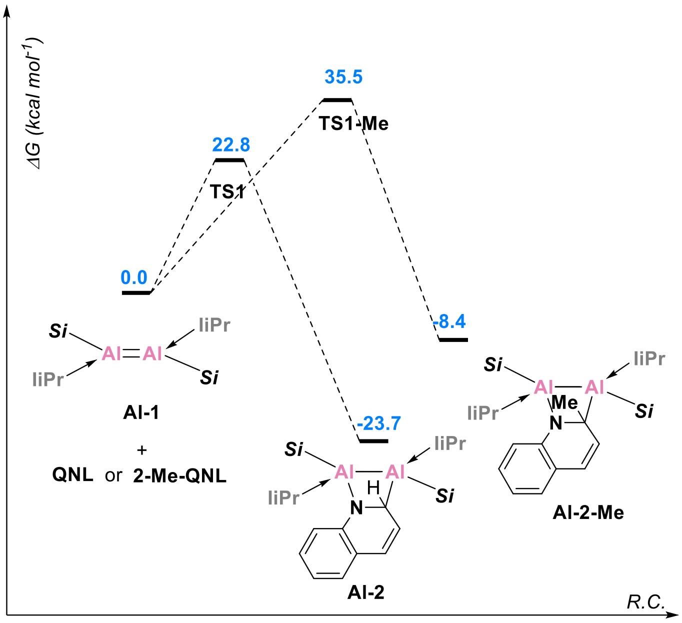

Supplementary Figure 58. Calculated reaction pathway for the reaction of **Al-1** with 2-Me quinoline.

4 Supplementary References

1. APEX suite of crystallographic software, APEX 4 version 2021.10-0; Bruker AXS Inc.: Madison, Wisconsin, USA (2021).

2. SAINT, Version 7.56a and SADABS Version 2008/1; Bruker AXS Inc.: Madison, Wisconsin, USA (2008).

3. Sheldrick, G. M. SHELXL-2014, University of Göttingen, Göttingen, Germany (2014).

4. Hübschle, C. B.; Sheldrick, G. M.; Dittrich, B. ShelXle: A Qt Graphical User Interface for SHELXL. *J. Appl. Cryst.* **44**, 1281-1284 (2011).

5. Sheldrick, G. M. SHELXL-97, University of Göttingen, Göttingen, Germany (1998).

6. Wilson, A. J. C. International Tables for Crystallography, Vol. C, Tables 6.1.1.4 (pp. 500–502), 4.2.6.8 (pp. 219–222), and 4.2.4.2 (pp. 193–199); Kluwer Academic Publishers: Dordrecht, The Netherlands (1992).

7. Macrae, C. F.; Bruno, I. J.; Chisholm, J. A.; Edgington, P. R.; McCabe, P.; Pidcock, E.; Rodriguez-Monge, L.; Taylor, R.; van de Streek, J.; Wood, P. A. Mercury CSD 2.0 - new features for the visualization and investigation of crystal structures. *J. Appl. Cryst.* **41**, 466–470 (2008).

8. Neese, F. Software update: The ORCA program system—Version 5.0. *WIREs Comput. Mol. Sci.***12**, e1606 (2022).

9. Grimme, S.; Hansen, A.; Ehlert, S.; Mewes, J. M. r^{2}SCAN-3c: A "Swiss army knife" composite electronic-structure method. *J. Chem. Phys.* **154**, 064103 (2021).

10. Furness, J. W.; Kaplan, A. D.; Ning, J.; Perdew, J. P.; Sun, J. Correction to “Accurate and Numerically Efficient r2SCAN Meta-Generalized Gradient Approximation”. *J. Phys. Chem. Lett.* **11**, 9248 (2020).

11. Furness, J. W.; Kaplan, A. D.; Ning, J.; Perdew, J. P.; Sun, J. Accurate and Numerically Efficient r2SCAN Meta-Generalized Gradient Approximation. *J. Phys. Chem. Lett.* **11**, 8208–8215 (2020).

12. Kruse, H.; A geometrical correction for the inter- and intra-molecular basis set superposition error in Hartree-Fock and density functional theory calculations for large systems. *J. Chem. Phys.* **136**, 154101 (2012).

13. Caldeweyher, E.; Bannwarth, C.; Grimme, S. Extension of the D3 dispersion coefficient model. *J. Chem. Phys.* **147**, 034112 (2017).

14. Caldeweyher, E.; Ehlert, S.; Hansen, A.; Neugebauer, H.; Spicher, S.; Bannwarth, C.; Grimme, S. A generally applicable atomic-charge dependent London dispersion correction. *J. Chem. Phys.* **150**, 154122 (2019).

15. Caldeweyher, E.; Mewes, J. M.; Ehlert, S.; Grimme, S. Extension and evaluation of the D4 London-dispersion model for periodic systems. *Phys. Chem. Chem. Phys.* **22**, 8499–8512 (2020).

16. Marenich, A. V.; Cramer, C. J.; Truhlar, D. G. Universal Solvation Model Based on Solute Electron Density and on a Continuum Model of the Solvent Defined by the Bulk Dielectric Constant and Atomic Surface Tensions. *J. Phys. Chem. B* **113**, 6378–6396 (2009).

17. Zhao, Y.; Truhlar, D. G. Design of Density Functionals That Are Broadly Accurate for Thermochemistry, Thermochemical Kinetics, and Nonbonded Interactions. *J. Phys. Chem. A* **109**, 5656–5667 (2005).

18. Weigend, F.; Ahlrichs, R. Balanced basis sets of split valence, triple zeta valence and quadruple zeta valence quality for H to Rn: Design and assessment of accuracy. *Phys. Chem. Chem. Phys.* **7**, 3297–3305 (2005).

19. Weigend, F. Accurate Coulomb-fitting basis sets for H to Rn. *Phys. Chem. Chem. Phys.* **8**, 1057–1065 (2006).

20. Hellweg, A.; Hättig, C.; Höfener, S.; Klopper, W. Optimized accurate auxiliary basis sets for RI-MP2 and RI-CC2 calculations for the atoms Rb to Rn. *Theor. Chem. Acc.* **117**, 587–597 (2007).
